# Label-Free Proteomic Analysis of Smoke-Drying and Shade-Drying Processes of Postharvest Rhubarb: A Comparative Study

**DOI:** 10.3389/fpls.2021.663180

**Published:** 2021-05-26

**Authors:** Wei Liang, Yuan Chen, Xia Li, Fengxia Guo, Jiachen Sun, Xuemin Zhang, Bo Xu, Wenyuan Gao

**Affiliations:** ^1^Gansu Provincial Key Lab of Arid Land Crop Science, College of Agronomy, College of Life Science and Technology, Gansu Agricultural University, Lanzhou, China; ^2^School of Pharmaceutical Science and Technology, Tianjin University, Tianjin, China; ^3^School of Biotechnology and Food Science, Tianjin University of Commerce, Tianjin, China; ^4^Key Laboratory of Modern Chinese Medicine Resources Research Enterprises, Tianjin, China

**Keywords:** rhubarb, smoke-drying, shade-drying, label-free proteomic **analyses**, postharvest drying process

## Abstract

Postharvest processing plays a very important role in improving the quality of traditional Chinese medicine. According to previous studies, smoke-drying could significantly promote the accumulation of the bioactive components and pharmacological activities of rhubarb, but so far, the molecular mechanism has not been studied yet. In this research, to study the molecular mechanisms of postharvest processing for rhubarb during shade-drying and smoke-drying, label-free proteomic analyses were conducted. In total, 1,927 differentially abundant proteins (DAPs) were identified from rhubarb samples treated by different drying methods. These DAPs were mainly involved in response and defense, signal transduction, starch, carbohydrate and energy metabolism, and anthraquinone and phenolic acid biosynthesis. Smoke-drying significantly enhanced the expression of proteins involved in these metabolic pathways. Accordingly, the molecular mechanism of the accumulation of effective ingredients of rhubarb was clarified, which provided a novel insight into the biosynthesis of active ingredients that occur during the rhubarb dry process.

## Introduction

Rhubarb is promoted as a remarkable and ancient plant that is widely used in Chinese herbal medicine (Wang et al., 2018); it was exported from China and is still used as a vital agent for various medical procedures (Kalisz et al., 2020). Rhubarb is now distributed worldwide and is used as both a food and medicine. *Rheum palmatum* L., *Rheum tanguticum* Maxim. ex Balf., and *Rheum officinale* Baill’s rhizomes are commonly used as medicinal rhubarb in China, while *Rheum rhaponticum* L.’s petioles are used as a vegetable in Europe (Romaniello, 2016; Kalisz et al., 2020). Fresh rhubarb cannot be used as a medicinal material immediately after harvest; it needs to undergo a series of processes to make it suitable for use as a decoction or as raw material in Chinese medicines. Smoke-drying is recognized as the traditional postharvest method, with rhubarb placed in a smoke-filled drying room for 2–3 months (Sun et al., 2018). In China’s primary producing area of rhubarb, Li County, Gansu Province, smoke-drying is extensively used for rhubarb and Angelica, and here it is considered to be a critical step in the postharvest processing. Smoke-drying of medicinal plants is a complicated process. Generally speaking, smoke refers to the mixture of air, carbon dioxide, water vapor, methane, and other gaseous components and liquid or solid components with a diameter of 0.08–0.15 μm. Smoke-drying is a process in which the compounds in the smoke are continuously deposited on the surface of a plant and from there migrate into the plant tissues (Simko, 2005). The accumulation of these compounds in plant tissues stimulates a corresponding stress response, thereby affecting the synthesis of secondary metabolites.

As indicated by modern research, freshly harvested plant materials still have physiologically active organs; therefore, postharvest plant organs can undergo a series of physiological and biochemical changes during the drying process. These variations may cause further changes in plant metabolism and biosynthesis, including the production of new metabolites or the elevation of secondary metabolite content (Briskin, 2000; Owiti et al., 2011; Kim et al., 2016). In recent years, researchers have found that postharvest processing can affect the synthesis of bioactive compounds. It is suggested that fresh *Artemisiae argyi Folium* contains traces of phenolic acids and flavonoids, whereas numerous hydrophilic compounds are detected in the dried state (Li et al., 2019). Therefore, speculation began that hydrophilic compounds are synthesized during postharvest processes. It has been noted that postharvest drying has a significant impact on the coumarin, phenoloid, and essential oil content of *Notopterygium franchetii* (Su et al., 2019). Previous studies found that salvianolic acid B content increases significantly during the postharvest drying process of Danshen (Li et al., 2012). The latter study concludes that postharvest drying expedites the senescence process, thereby changing the synthesis of bioactive components and the accumulation of secondary metabolites. Accordingly, carefully planned postharvest processing methods are critical to improving the quality of medicinal plants.

Phenolic acid, sennoside, and anthraquinone are the major active substances in rhubarb that are synthesized during secondary metabolism (Sun et al., 2017; Xiong et al., 2019). An accumulation of secondary metabolites is commonly promoted after plant stress (Isah, 2019). Therefore, environmental stressors, such as pathogen attack, UV irradiation, salinity, drought, and temperature, often lead to an increased accumulation of secondary metabolites (Sharma et al., 2019). In addition, nutrient stress significantly impacts phenolic levels in plant tissues (Chalker-Scott and Fuchigami, 1989). It is reported that the formation of phenyl amides and strong accumulation of polyamines in beans and tobacco as a response to abiotic stresses suggest that external stress could significantly affect secondary metabolism (Edreva et al., 2007). Similarly, anthocyanin accumulation is known to take place following a string of environmental stresses, such as UV, blue light, high-intensity light, wounding, pathogen attack, drought, and sugar, and nutrient deficiency (Winkel-Shirley, 2001). Salt stress is usually a creator of both ionic and osmotic stresses in plants, which leads to a multiplication or reduction of specified secondary metabolites in plants (Mahajan and Tuteja, 2005). Moreover, an elevation of polyphenol content has been noted under salt stress in several plants (Parida and Das, 2005). Accordingly, plant secondary metabolites are highly affected by adaptation to an environment and defense against external stress factors.

Reactive oxygen species (ROS) are endogenously synthesized by several plant organelles (e.g., chloroplasts, mitochondria, and peroxisomes) and have multiple roles in plant metabolism (for example, cellular messengers, and redox regulators) (Raja et al., 2017). In normally growing plants, ROS production and elimination are maintained in a balanced state (Gilroy et al., 2016). However, external abiotic stressors can break the balance between ROS and antioxidant enzymes (Mignolet-Spruyt et al., 2016). Therefore, under a series of abiotic stressors, plant cells will produce a large amount of ROS. These ROS are signaling molecules that indirectly or directly affect the physiological activities of plant cells, prompting plants to respond to external stressors (Dietz, 2015, 2016; Dietz et al., 2016). In addition, the disruption of the balance between ROS and antioxidant enzymes also affects the secondary metabolism of plants. Therefore, antioxidant enzyme system not only can transmit signals within cells but also regulate the synthesis of secondary metabolites in plants.

According to existing studies, smoke-drying promotes the accumulation of bioactive components and pharmacological activities in rhubarb (Sun et al., 2018). During smoke-drying, rhubarb is subject to a series of biotic and abiotic stresses (namely, drought, hypoxia, damage, and bacterial infection). When rhubarb is impacted by abiotic stress and aging, the dynamic balance of ROS in plants is broken, the activity of antioxidant enzymes varies, and the production of secondary metabolites is further affected. However, the underlying reasons for the accumulation of secondary metabolites during the smoke-drying process are still not completely understood. For the first time, to our knowledge, this study identified, characterized, and quantitated proteomics information during the smoke-drying and shade-drying processes of rhubarb with the purpose of investigating the molecular mechanisms of the drying process. The simple and integrative approach here was to derive and understand the molecular variations involved in the postharvest handling of rhubarb that are important for advancing medicinal plant research.

## Materials and Methods

### Plant Material and Experimental Design

Rhubarb (*R. palmatum* Linn.) rhizomes were harvested from Li County, Gansu Province, China, on November 1, 2019, at the withered of ground parts and were treated on the day when rhubarb was transported to the laboratory. Before drying the rhubarb, the lateral roots and outer skin were cut off, and then the rhizomes with a uniform shape and size were selected as experimental materials.

Twenty-seven rhubarbs were chosen and divided into three groups. Nine independent pooled samples were assembled and considered as control sample (fresh, X), and other rhubarbs were further subjected to different postharvest processing for shade-drying and smoke-drying, respectively. Each treatment consisted of nine rhubarbs, which were divided into three replicates. The shade-drying treatment (K) put rhubarb into a cool and ventilated place at a temperature of 10–12°C. The smoke-drying treatment (Y) was simulating the rhubarb traditional drying method. Through previous experiments, we found that the accumulation of effective ingredients in rhubarb has the most obvious promotion effect when it is smoke-drying at a concentration of 25%. Therefore, in this experiment, we put the rhubarb sample in 25% smoke for smoke-drying. The smoke generated from applewood and its main gas ratio is approximately N_2_:O_2_:CO_2_:CO = 79:15:4.7:0.3. In brief, store the rhubarb in a closed gas box and pass the smoke with a concentration of 25% into the box with a flow velocity of 0.5 m/s, at 15–17°C.

Plant tissues will gradually lose their activity during the drying process. In order to better research the changes of various protein during the drying process of rhubarb, we choose to sample after 4 days of treatment (smoke-drying and shade-drying rhubarb has 53.84 ± 1.78 and 54.02 ± 0.39% water content). The samples collected from different dry methods were immediately mixed and frozen in liquid nitrogen and stored on the condition of −80°C until further analysis.

### Metabolite Analyses

Rhubarbs are complex mixtures containing various kinds of constituents. Therefore, we chose to measure 16 active ingredients in rhubarb including four polyphenols [gallic acid (1), catechins (2), epicatechin (3), and epicatechin gallate (4)], two anthrones [sennoside B (5) and sennoside A (6)]; 5 free anthraquinones [aloe-emodin (7), rhein (8), emodin (9), chrysophanol (10), and physcion (11)], and five combined anthraquinones [aloe emodin 8-β-D-glucoside (12), rhein 8-β-D-glucoside (13), emodin 8-β-D-glucoside (14), chrysophanol 8-β-D-glucoside (15), and physcion 8-β-D-glucoside (16)].

#### Sample Preparation

Lyophilized tissue was ground and sieved through a 60-mesh screen. The sample (2 g) powder was extracted with 80% methanol (20 mL) upon 50-min ultrasonication at 60°C.

#### Preparation of the Standard Solution

Standard solution was prepared by dissolving 16 standard reference substances in 10 mL of ethanol/dimethyl sulfoxide (0.5:0.5, vol/vol). Then, the standard solution was stored in the refrigerator at 4°C.

#### HPLC-PDA System

Shimadzu i-Series HPLC system was used to quantitative analysis (Shimadzu, Japan) and comprised a binary solvent delivery pump, a PDA detector, and a Kromasil C_18_ column (4.6 × 250 mm, 100 Å, 5 μm) maintained at 40°C at a flow rate of 1.0 mL/min. The mobile phase contained 2% formic acid aqueous solution (A) and acetonitrile (B) with the following gradient program: 0–5 min, 3–6% B; 5–20 min, 6–6% B; 20–35 min, 6–9% B; 35–45 min, 9–12% B; 35–45 min, 9–12% B; 45–80 min, 12–14% B; 80–100 min, 14–19% B; 100–130 min, 19–33% B; 130–155 min, 33–55% B; and 155–185 min, 55% B. The injection volume was 10 μL, and the detection wavelength was 280 nm.

### Superoxide Dismutase, Peroxidase, Catalase, and Ascorbate Peroxidase Activity Assay

The 1.00 g rhubarb fresh tissue powder was mixed with 9 mL normal saline for enzymatic extraction. The solution was centrifuged on the condition of 4°C, 2,500 revolutions/min lasting 10 min; besides, the supernatant was gathered for determining superoxide dismutase (SOD), peroxidase (POD), catalase (CAT), and ascorbate peroxidase (APX) activity. The assay kits were bought out of Nanjing Jiancheng Bioengineering Institute.

### Protein Extraction and Filter-Aided Sample Preparation

Fresh, shade-drying, and smoke-drying rhubarb samples (2 g) were ground in liquid nitrogen. Proteins were extracted from 1 g of the resulting power as described by literature (Di Carli et al., 2011). The eventually dried protein pellet was stored at −80°C until use. The dried protein pellet was dissolved in lysis buffer comprising 7 M urea, 2 M thiourea, and 5% CHAPS, as well as 2 mM tributyl phosphine. The dissolved protein solution was centrifuged again at 12,000 × *g* for 10 min at 4°C, and the supernatant was collected as final protein sample. Protein concentration was determined by using the Bradford method with bovine serum albumin as standard (Bradford, 1976).

Proteins were reduced with DTT (final concentration of 20 mM) at 45°C for 30 min. Then, the samples were alkylated with 50 mM IAA (iodoacetamide) and kept in dark environment at room temperature for 1 h. All the samples put into the ultrafiltration tube and centrifugation were made for 15 min, and then 100 μL of 50 mM NH_4_HCO_3_, and centrifuge was made lasting 10 min, with repetition of three times. Eventually, digestion of proteins was made in a new tube based on 1:50 trypsin/protein ratio on the condition of 37°C lasting 12 h. Digested fractions were collected and stored at −80°C for mass spectrometry (MS) analysis.

### Protein Identification by Mass Spectrometer

The loading of peptides in 0.1% formic acid was made on to a Fusion Lumos mass spectrometer, as well as an Easy-nLC 1200 system (Thermo Fisher Scientific), which is set with a 150 μm × 2 cm self-packed C18 trap column (particle size 3 μm, Dr. MASCH GmbH, Germany) based on the isolation on a 150 μm × 30 cm self-packed C18 analytical column (the particle size was 1.9 μm, Dr. MASCH GmbH).

The elution and separation of peptides were made out of the trap column by employing 0.1% formic acid (mobile phase A); 80% acetonitrile is made up of 0.1% formic acid (mobile phase B). Gradients were operated from 8 to 12% B over 10 min, 12 to 27% over 69 min, 27 to 45% over 28 min, 45 to 95% over 3 min, and 95% over 10 min. The mass spectrometer was conducted in the data-dependent acquisition mode by utilizing Xcalibur 4.0 software. Besides, a single full-scan mass spectrum was used in the Orbitrap (350–1,800 m/z, 120,000 resolution) previous to data-dependent MS2 scans at the rate of 30% collision energy (HCD) within an ion trap.

### Bioinformatics Analysis

The search criteria were as follows: full tryptic specificity was required; two missed cleavages were allowed; carbamidomethylation (C) was set as fixed modification; oxidation (M) was set as dynamic modifications; precursor ion mass tolerance was 20 ppm for all MS acquired in the Orbitrap mass analyzer, and fragment ion mass tolerance was 0.6 Da for all MS2 spectra acquired in the ion trap. High confidence score filter (false discovery rate <1%) was used to select the “hit” peptides, and their corresponding MS/MS spectra were manually inspected. Proteins with fold changes ≥1.2 or ≤0.833 and *P* < 0.05 were selected as differentially abundant proteins (DAPs) (Brosch et al., 2009). The GO database^1^ can be used for functional annotation of DAPs. The Clusters of Orthologous Groups (COG) database^2^ and the Kyoto Encyclopedia of Genes and Genomes (KEGG) database^3^ were used to classify and group the DAPs.

### Statistical Analysis

All data are presented as means ± standard error (SE). The degree of freedom between groups is 8. The data of physical and chemical index were analyzed by one-way analysis of variance (ANOVA) and Duncan tests with SPSS version 19.0. All figures were processed and analyzed using Origin 8.0 software.

## Results

### Effect of Dry Method on the 16 Chemical Compositions of Rhubarb

**FIGURE 1:**
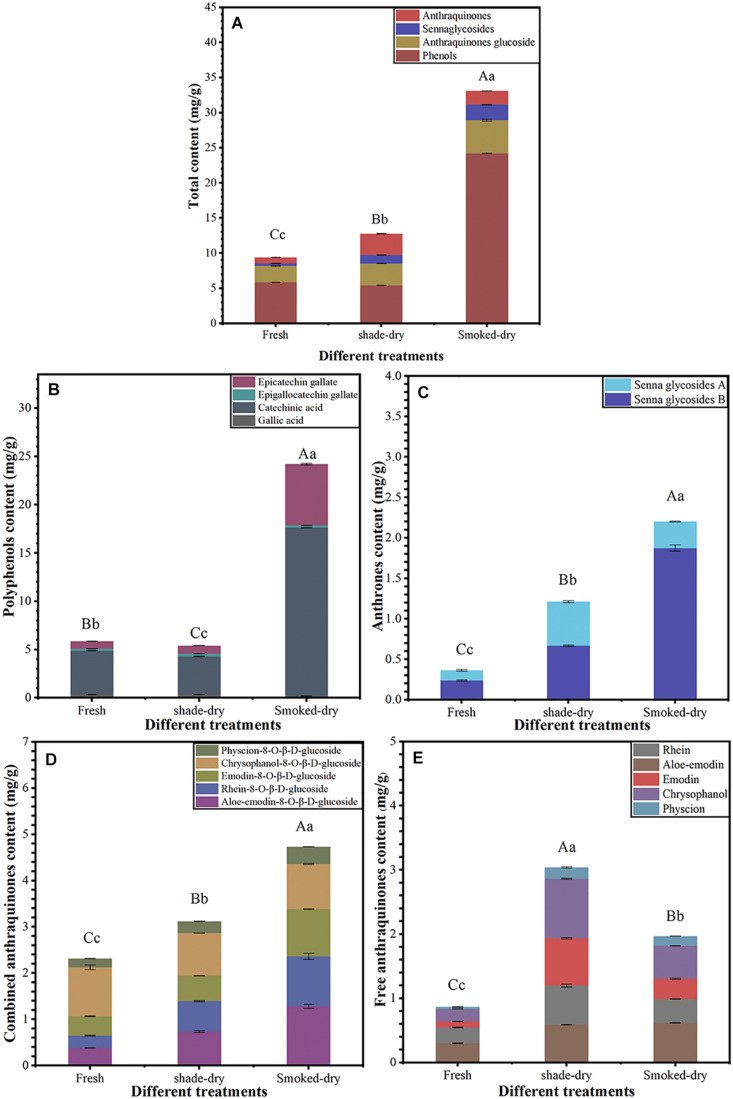
Sixteen active ingredient analysis of fresh rhubarbs, shade-drying, and smoke-drying. **(A)** The total content of active ingredients at the different dry method, **(B)** polyphenols content, **(C)** anthrones content, **(D)** combined anthraquinones content, **(E)** free anthraquinones content. The error bars represent the means ± SE (*n* = 3). The degree of freedom between groups is 8. Perform ANOVA on total content, polyphenols content, anthrones content, combined anthraquinones content, and free anthraquinones content, respectively. Different letters indicate a significant difference (capital letters *p* < 0.01, small letters *p* < 0.05).

As shown in Figure 1, the change in active ingredients after a 4-day drying period was significant (*p* < 0.01). The total content of 16 active ingredients increased by 35.98 and 252.21% after 4 days of shade-drying and smoke-drying, respectively (Figure 1A). The polyphenol content in smoke-drying rhubarb was 24.21 mg/g, nearly 3.13-fold higher than fresh (5.86 mg/g; Figure 1B). The contents of anthrones in shade-drying (1.21 mg/g) and smoke-drying (2.20 mg/g) rhubarb were both significantly higher than that in fresh rhubarb (0.36 mg/g; Figure 1C). Combined anthraquinone contents in shade-drying and smoke-drying rhubarb were 3.12 and 4.73 mg/g, respectively, and were significantly higher than in fresh rhubarb (2.31 mg/g). Free anthraquinone contents in shade-drying and smoke-drying were 3.04 and 1.97 mg/g, respectively, and were also significantly higher than in fresh rhubarb (0.87 mg/g; Figure 1D). These results show that both shade-drying and smoke-drying could increase effective ingredient content of rhubarb, but the effect of smoke-drying was more obvious.

### Effect of Dry Method on SOD, POD, CAT, and APX Activity

**FIGURE 2:**
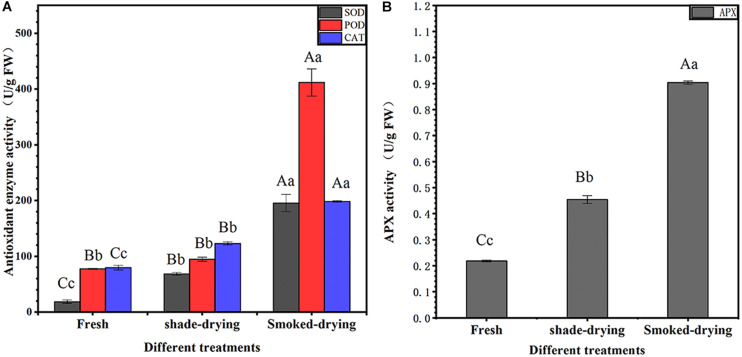
Activity of antioxidant-related enzymes. **(A)** SOD, POD, and CAT activity; **(B)** APX activity. The error bars represent the means ± SE (*n* = 3). The degree of freedom between groups is 8. Perform ANOVA on SOD, POD, CAT, and APX activities, respectively. Different letters indicate a significant difference (capital letters *p* < 0.01, small letters *p* < 0.05).

The changes in SOD, POD, CAT, and APX are shown in Figure 2. SOD activity was increased by 270.96 and 962.19% after 4 days of shade-drying and smoke-drying, respectively. POD activity was increased by 22.23 and 431.50% after 4 days of shade-drying and smoke-drying, respectively. CAT activity was increased by 54.61 and 149.34% after 4 days of shade-drying and smoke-drying, respectively. Finally, APX activity was increased by 108.00 and 313.68% after 4 days of shade-drying and smoke-drying, respectively. SOD, POD, CAT, and APX are important enzymes involved in the elimination of ROS in organisms. The significant increase in the activity of these enzymes in smoke-drying rhubarb indicated that this process produced more intense external stress and higher levels of ROS.

### Protein Identification and Quantification

**FIGURE 3:**
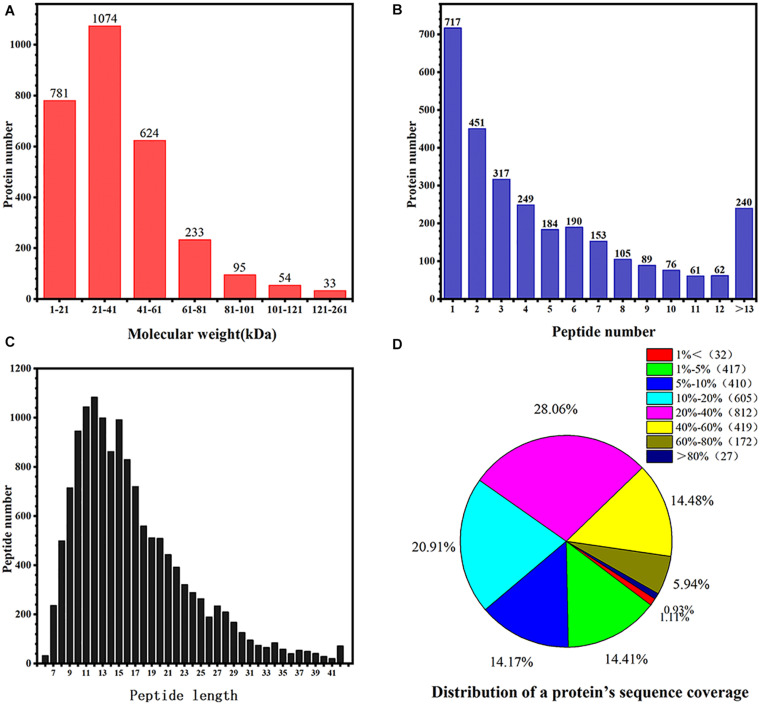
Identification and analysis of the proteome of rhubarb. **(A)** Protein molecular weight in rhubarb; **(B)** numbers of peptide identified into the rhubarb proteins; **(C)** the percentage of different peptide lengths in total amino acids. **(D)** Distribution of a protein’s sequence coverage.

In total, this research covered 153,237 spectra, which obtained identification out of rhubarb, on which dry process was, and 556,720 of these spectra conforming to known spectra. Additionally, there are in total 3,439 proteins based on the lowest one unique peptide that received the identification out of all the rhubarb samples. The distribution of protein molecular weight is summarized in Figure 3A. The molecular weights of most proteins were between 1 and 100 kDa, with only a few greater than 101 kDa. Figure 3B expresses the peptide identification numbers of proteins and shows that the number of peptides in the identified proteins was mostly between 1 and 12. Figure 3C shows the distribution of the identified peptide lengths. The results revealed that the number of amino acids in the peptides was mostly between 8 and 20. The distribution of protein sequence coverage is summarized in Figure 3D, where 77.62% of protein sequence coverage was between 5 and 60%. This indicated that the proteomics data detected were of high quality.

**FIGURE 4:**
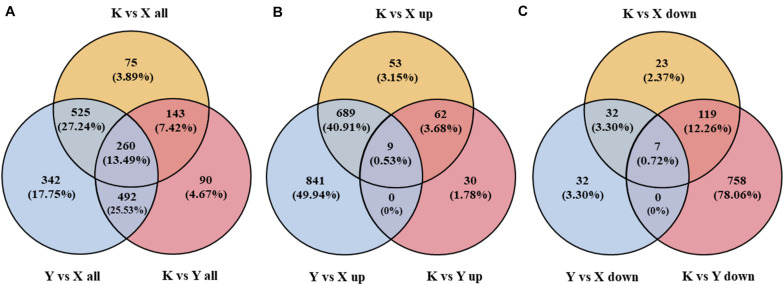
Venn diagram of DAPs identified from different comparisons in rhubarb samples. **(A)** Venn diagram of all the DAPs identified in rhubarb samples; **(B)** Venn diagram of the up-regulated proteins identified in rhubarb samples; **(C)** Venn diagram of the down-regulated proteins identified in rhubarb samples.

In the current study, 1,003 (822 up, 181 down), 1,619 (1,548 up, 71 down), and 985 (101 up, 884 down) DAPs were obtained from the comparisons of shade-drying rhubarb vs. fresh rhubarb, smoke-drying rhubarb vs. fresh, and shade-drying rhubarb vs. smoke-drying rhubarb, respectively. In total, 1,927 proteins were detected in this research; 260, 1,190, and 507 proteins showed significant differences in three, two, and one comparison, respectively (Figure 4A). Among the 1,684 up-regulated (971 down-regulated) proteins, 9 (7), 3 (151), and 760 (813) proteins were identified in three, two, and one comparison, respectively (Figures 4B,C). The figure of up-regulated proteins was higher than for down-regulated proteins, showing that the drying process promoted the up-regulation of many proteins in rhubarb.

### Functional Classification and Annotation of DAPs

**FIGURE 5:**
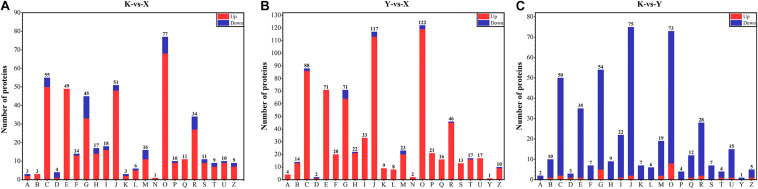
Functional classification of DAPs by COG database. **(A)** Functional classification of DAPs identified in shade-drying vs. fresh. **(B)** Smoke-drying vs. fresh. **(C)** Shade-drying vs. Smoke-drying.

According to COG analysis, DAPs were classified into 23 clusters. Posttranslational modification, protein turnover, chaperones; energy production and conversion; translation, ribosomal structure, and biogenesis; amino acid transport and metabolism; carbohydrate transport and metabolism; general function prediction only; and lipid transport and metabolism were the main functional categories identified from three different comparisons (Figure 5). In addition, there were no DAPs involved in nuclear structure and cell motility in shade-drying vs. fresh and shade-drying vs. smoke-drying, respectively (Figures 5A,B). Moreover, the numbers of down-regulated DEPs involved in shade-drying vs. smoke-drying were significantly higher than up- regulated ones (Figure 5C). However, the figures of up-regulated DAPs involved in shade-drying vs. fresh and smoke-drying vs. fresh were significantly higher than for down-regulated. We also found that the figures of up-regulated DAPs related to shade-drying vs. fresh and smoke-drying vs. fresh were significantly higher than for down-regulated DAPs. These results suggest that the drying process promoted the up-regulation of most proteins in rhubarb, and the up-regulation of DAPs was more sensitive to the smoke-drying process.

**FIGURE 6:**
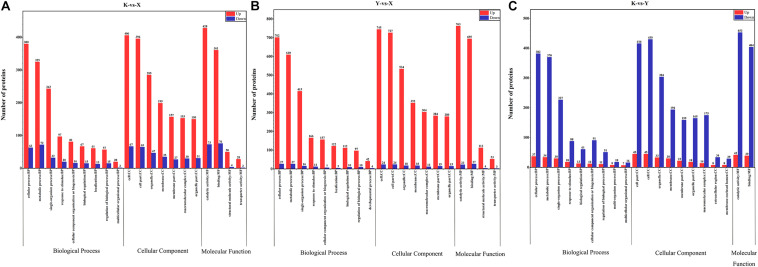
Bioinformatics analysis of DAPs through GO in postharvest change processes in rhubarb. **(A)** Functional annotation of DAPs identified in shade-drying vs. fresh. **(B)** Smoke-drying vs. fresh. **(C)** Shade-drying vs. smoke-drying.

The Gene Ontology (GO) database was used to annotate all DAPs for different drying methods. The top 20 GO functional annotation analyses are shown in Figure 6. GO annotation concerning shade-drying vs. fresh assignment was made to nine biological process categories (cellular process, metabolic process, single-organism process, response to stimulus, cellular component organization, or biogenesis, biological regulation, localization, regulation of biological process, and multicellular organismal process), seven cellular component categories (cell, cell part, organelle, membrane, membrane part, macromolecular complex, and organelle part), and four molecular function categories (catalytic activity, binding, structural molecule activity, and transporter activity; Figure 6A). As for the GO annotation concerning smoke-drying vs. fresh, assignment was made to nine biological process categories (cellular process, metabolic process, single-organism process, response to stimulus, cellular component organization, or biogenesis, localization, biological regulation, regulation of biological process, and developmental process), seven cellular component categories (cell, cell part, organelle, membrane, macromolecular complex, membrane part, and organelle part), and four molecular function categories (catalytic activity, binding, structural molecule activity, and transporter activity; Figure 6B). GO annotation concerning the shade-drying vs. smoke-drying assignment was made to nine biological process categories (cellular process, metabolic process, single-organism process, response to stimulus, biological regulation, cellular component organization, or biogenesis, regulation of biological process, multiorganism process, and multicellular organismal process), nine cellular component categories (cell part, cell, organelle, membrane, membrane part, organelle part, macromolecular complex, extracellular region, and membrane-enclosed lumen), and two molecular function categories (catalytic activity, binding; Figure 6C). The number of up-regulated proteins in shade-drying and smoke-drying rhubarb was significantly higher than for fresh. According to GO analysis of the DAPs, the three comparisons revealed that cellular process, metabolic process, single-organism process, cell part, cell, organelle, catalytic activity, and binding might be closely related to postharvest change processes in rhubarb.

### Functional Classification and Annotation of DAPs

**FIGURE 7:**
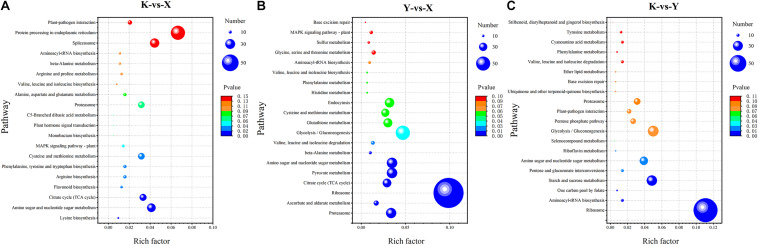
Scatterplot of the KEGG pathway enrichment statistics of DAPs. **(A)** Shade-drying vs. fresh. **(B)** Smoke-drying vs. fresh. **(C)** Shade-drying vs. smoke-drying.

To further investigate the biochemical pathways during rhubarb drying, all DAPs were mapped to the KEGG database. There were 749, 1241, and 756 DAPs obtained from shade-drying vs. fresh, smoke-drying vs. fresh, and shade-drying vs. smoke-drying, and these were mapped onto 102, 107, and 102 pathways, respectively, and seven, nine, and seven pathways were significantly enriched. The top 20 KEGG pathways obtained from the three comparisons are shown in Figure 7. The significantly enriched pathways in shade-drying vs. fresh were lysine biosynthesis, amino sugar and nucleotide sugar metabolism, citrate cycle (TCA cycle), flavonoid biosynthesis, arginine biosynthesis, phenylalanine, tyrosine and tryptophan biosynthesis, and cysteine and methionine metabolism (Figure 7A). The significantly enriched pathways in smoke-drying vs. fresh were proteasome; ascorbate (AsA) and aldarate metabolism; ribosome; citrate cycle (TCA cycle); pyruvate metabolism; amino sugar and nucleotide sugar metabolism; β-alanine metabolism; valine, leucine, and isoleucine degradation; and glycolysis/gluconeogenesis (Figure 7B). Moreover, the significantly enriched pathways in shade-drying vs. smoke-drying were ribosome, aminoacyl-tRNA biosynthesis, one-carbon pool by folate, starch and sucrose metabolism, pentose and glucuronate interconversions, amino sugar and nucleotide sugar metabolism, and riboflavin metabolism (Figure 7C). These biochemical metabolism pathways will help us to further understand the physiological changes of rhubarb during the drying process.

## Discussion

Rhubarb undergoes a series of physiological changes during the drying process, and these changes will further affect the metabolism and synthesis of endogenous chemicals. Aiming to accurately detect the changes that take place, we determined the content of 16 active ingredients and antioxidant enzyme activities throughout the early stage of drying. The test results showed that after 4 days of drying, the contents of active ingredients in rhubarb and the activity of antioxidant enzymes were significantly higher than for fresh samples. Therefore, choosing to perform proteomics measurements at 4 days after the rhubarb has started to dry can accurately clarify the changes undergone during the drying process. In this study, 1,927 DAPs were identified. These DAPs were mainly related to carbohydrate and energy metabolism (TCA cycle, starch and sucrose metabolism, glycolysis/gluconeogenesis, etc.), secondary metabolites (flavonoid biosynthesis, shikimic acid biosynthesis, etc.), signal transduction [mitogen-activated protein kinase (MAPK) signaling pathway, phosphatidylinositol signaling system, plant hormone signal transduction, etc.], and stress response and defense (proteasome, plant–pathogen interaction, endocytosis, etc.). Through bioinformatics and biochemical analyses, we verified that the changes that took place in the active ingredients of rhubarb during smoking or shade drying were the result of an interaction among multiple stress factors during the drying process.

### Proteins Related to Signal Transduction

**FIGURE 8:**
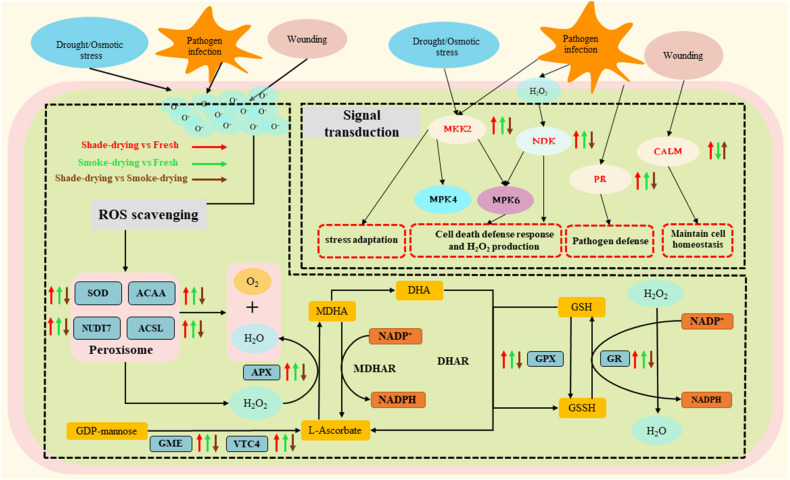
Overview of the important differentially expressed proteins of the stress response and signal transduction involved in rhubarb during the dry process. The arrow up represents the up-regulation of proteins. The arrow down represents the down-regulation. MKK2, mitogen-activated protein kinase kinase; NDK, nucleoside diphosphate kinase; PR, pathogenesis-related protein 1; CALM, calmodulin; SOD, superoxide dismutase; ACAA, acetyl-CoA acyltransferase; NUDT7, peroxisomal coenzyme A diphosphatase NUDT7; ACSL, long-chain acyl-CoA synthetase; APX, L-ascorbate peroxidase; GPX, glutathione peroxidase; GR, glutathione reductase; GME, GDP-D-mannose 3′,5′-epimerase; VTC4, inositol-phosphate phosphatase; MDHAR, MDHA reductase; DHAR, dehydroascorbate reductase; MDHA, monodehydroascorbate; DHA, dehydroascorbate; GSH, reduced glutathione; GSSH, oxidized glutathione; NADP^+^/NADPH, nicotinamide adenine dinucleotide phosphate.

A vital characteristic of living plants is the ability to sense external environmental signals and respond to environmental variations (Zhong et al., 2017). Twenty-two DAPs involved in signal transduction mechanisms were identified (Supplementary Table 1). Ca^2+^ ions, as second messengers, exert a fundamental effect on a wide range of environmental responses (La Verde et al., 2018). Calmodulin (CALM) critically impacts signal transduction under heat stress, thus triggering a series of physiological responses (Owiti et al., 2011). As revealed in this research, CALM was elevated in shade-drying vs. fresh and declined in smoke-drying vs. fresh, respectively. These data suggest that shade-drying and smoke-drying had different influences on the expression levels of CALM (Figure 8).

In most eukaryotes, MAPK is an important step in environmental signal transduction (Xu and Zhang, 2015). Activated MAPK cascades are of vital importance for protecting plants against a variety of environmental stresses (Bi et al., 2018). The expression of 14 DAPs associated with MAPK cascades was suggested to be elevated. These DAPs might be involved in pathogen infection, pathogen attack, cold, drought, and wounding during the rhubarb-drying process.

In plants, pathogenesis-related protein 1 (PR-1) proteins are among the most abundantly produced proteins during defense responses. Furthermore, it is reported that external abiotic stimuli can significantly influence the expression of the PR-1 gene (Breen et al., 2017). Nucleoside diphosphate kinases (NDKs) are capable of catalyzing the reversibility-based transfer made by the terminal phosphate from the range of a donor nucleoside triphosphate toward an acceptor nucleoside diphosphate (NDP) (Dorion and Rivoal, 2018). NDP kinase is reported to be critical for cell death (Luzarowski et al., 2017). According to biochemical and genetic analyses in *Arabidopsis*, MAPK kinase 2 (MKK2) responds to diverse stressors including drought and temperature stress (Brader et al., 2007). As revealed in the present research, the expressions of PR-1, MKK2, and NDK were up-regulated in shade-drying vs. fresh and smoke-drying vs. fresh. As the results showed, the proteins might play a role in assisting rhubarb to resist environmental stress and delay death. However, the expressions of PR-1, MKK2, and NDK were down-regulated in shade-drying vs. smoke-drying, indicating that the smoke-drying process may exert stronger stress factors on rhubarb.

These findings showed that protein expressions related to signal transduction and against stress were strengthened in rhubarb during the drying process, which might be a manifestation of the delay of death in rhubarb during the drying process.

### Proteins Related to Stress Response and Defense

High ROS levels are toxic to cells, and they are capable of influencing normal metabolism through oxidative damage to lipids, proteins, and DNA (Farooq et al., 2009; Liu and Wang, 2012). Plants have shaped a complex antioxidant system to protect themselves against oxidative damage (e.g., antioxidant enzymes and metabolites). These systems include the AsA–glutathione (AsA–GSH) cycle and antioxidant enzyme system (e.g., SOD, POD, CAT, and APX) (Zhang and Shi, 2018). Furthermore, a coregulated antioxidant system could help improve antioxidant activity. The AsA–GSH cycle acts as an effective antioxidative system to detoxify excess ROS by maintaining the ratios of AsA/dehydroascorbate (DHA) and GSH/glutathione disulfide (GSSG) (Asada, 1999; Figure 8).

It is reported that the activities of APX, CAT, and POD in alfalfa roots are enhanced under drought stress (Cao et al., 2017; Zhang and Shi, 2018). Similar results were found in the current study where SOD, APX, CAT, and POD activities in the rhubarb were found to increase during shade-drying or smoke-drying for 4 days (Figure 2). It is noteworthy that SOD and POD enzyme activities under smoke-drying were found to be significantly higher than those under shade-drying in the current study. Here, 25 DAPs related to the peroxisome and AsA–GSH cycles were identified during rhubarb-drying processes, and most of the mentioned proteins were up-regulated (Supplementary Table 2).

SOD-scavenged O_2_^–^ works as a catalyzer of the disproportionation reaction of O_2_^–^ in the body for the production of O_2_ and H_2_O_2_. In this study, the expressions of SOD increased in shade-drying vs. fresh and smoke-drying vs. fresh, which was similar to the research results using *Bupleurum* under osmotic stress (Yang et al., 2019). According to previous reports, up-regulated expression of 3-ketoacyl-CoA thiolase 2 (ACCA), peroxisomal coenzyme A diphosphatase (NUDT7), and long-chain acyl-CoA synthetase (ACSL) enhances abiotic stress resistance in plants (Ge et al., 2007; Pye et al., 2010; Zhang et al., 2020). The current research revealed that the expressions of ACCA, NUDT7, and ACSL were up-regulated in shade-drying vs. fresh and smoke-drying vs. fresh. Meanwhile, GDP-D-mannose 3′,5′-epimerase (GME) and inositol-phosphate phosphatase (VTC4) act as vital enzymes in AsA biosynthesis, where they catalyze the conversion of GDP-mannose to ascorbate. In this study, the expression of the mentioned two enzymes was suggested to increase significantly during the drying process of rhubarb, facilitating the accumulation of ascorbate.

Ascorbate is one of the principal antioxidants in plants. Generally, ascorbate is oxidized to monodehydroascorbate (MDHA) by APX, which reduces H_2_O_2_ and produces H_2_O (Foyer and Noctor, 2011). Glutathione reductase (GR) can catalyze the decrease of GSSG toward the sulfhydryl form GSH, which is a key molecule in resistance to oxidative stress. Plant glutathione peroxidases (GPXs) are known as ubiquitous enzymes, demonstrating their presence in different plant tissues, compartments, and developmental phases (Yang et al., 2005, 2006). Under different biotic and abiotic stresses, GPX activity and plant tolerance will be promoted (Milla et al., 2003). In the current research, the expression of APX, GR, and GPX was up-regulated during the shade-drying vs. fresh or smoke-drying vs. fresh. The up-regulation of the mentioned three enzymes might mitigate various stresses and slow senescence during the rhubarb-drying process.

According to the above research results, the antioxidant enzyme system of rhubarb is promoted during the drying process, where rhubarb cells enter a fierce state of resistance against aging and death at the early stage of drying. This intense cellular activity may improve synthesis of the medicinally effective components of rhubarb.

### Proteins Related to Carbohydrate and Energy Metabolism

**FIGURE 9:**
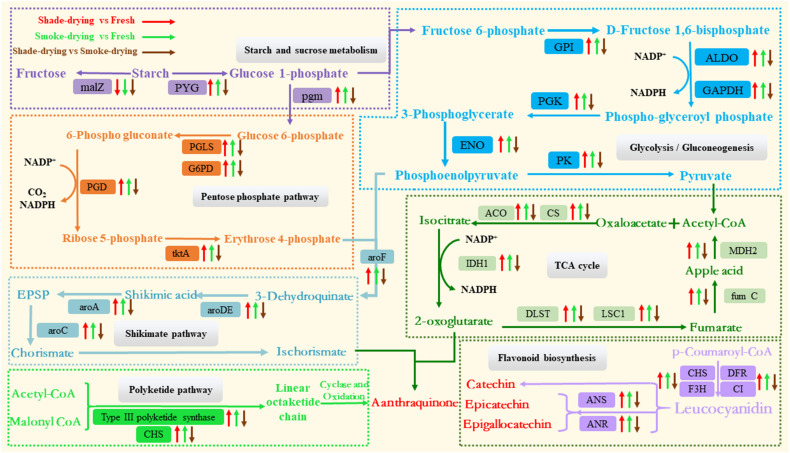
Overview of the important differentially expressed proteins of the main metabolic processes involved in rhubarb during the dry process. The arrow up represents the up-regulation of proteins. The arrow down represents the down-regulation. malZ, α-glucosidase; PYG, starch phosphorylase; pgm, phosphoglucomutase; PGLS, probable 6-phosphogluconolactonase; G6PD, glucose-6-phosphate 1-dehydrogenase; PGD, 6-phosphogluconate dehydrogenase; tktA, transketolase; GPI, phosphoglucose isomerase; ALDO, fructose-bisphosphate aldolase; GAPDH, glyceraldehyde-3 phosphate dehydrogenase; PGK, phosphoglycerate kinase; ENO, enolase; PK, pyruvate kinase; ACO, aconitate hydratase; CS, citrate synthase; IDH1, isocitrate dehydrogenase; DLST, 2-oxoglutarate dehydrogenase complex 1; LSC1, succinyl-CoA ligase; fumC, fumarate hydratase 1; MDH2, malate dehydrogenase; aroDE, shikimate dehydrogenase; aroA, 3-phosphoshikimate 1-carboxyvinyltransferase; aroC, chorismate synthase; CHS, chalcone synthase; DFR, dihydroflavonol-4-reductase; F3H, flavones 3-hydroxylase; CI, chalcone isomerase; ANS, anthocyanin synthase; ANR, anthocyanidin reductase.

Primary metabolism has the maximum number of differential proteins. In this research, we found 45, 55, 26, and 32 DAPs involved in starch and sucrose metabolism, glycolysis/gluconeogenesis, pentose phosphate pathway, and TCA cycle, respectively. Most of these differential proteins were up-regulated during the shade-drying or smoke-drying of rhubarb (Supplementary Table 3 and Figure 9).

Starch is the name given to the storage carbohydrate extensively identified in plants; it is composed of homopolymers of glucose (Streb and Zeeman, 2012). A reduction in starch levels has been demonstrated under various stressors (Thalmann and Santelia, 2017; Dong et al., 2018). Starch is degraded into glucose 1-phosphate by glycogen phosphorylase (PYG) and then forms glucose-6-phosphate after impaction by phosphoglucomutase (pgm). It is also degraded into fructose by α-glucosidase (malZ). The down-regulated expression of malZ and up-regulated expression of PYG during the shade-drying vs. fresh or smoke-drying vs. fresh processes showed that the drying process could accelerate the consumption of starch and produce glucose 1-phosphate. However, the expression of pgm and PYG were down-regulated in shade-drying vs. smoke-drying, indicating that the degradation of rhubarb starch was more active under conditions of smoke-drying. Glucose 1-phosphate from the degradation of sucrose and starch acts as a substrate for PPP and glycolysis/glycolysis pathways and promotes these metabolic pathways.

The PPP, which is widely found in plants, is an indispensable cellular metabolic pathway that exerts critical effects upon plant growth and development and also in response to biotic and abiotic stresses (Kruger and von Schaewen, 2003; Stincone et al., 2015). 6-Phosphogluconolactonase (PGLS), glucose-6-phosphate dehydrogenase (G6PD), 6-phosphogluconate dehydrogenase (PGD), and ribose-5-phosphate isomerase (tktA) are key enzymes in the PPP that oxidize glucose-6-phosphate to produce erythrose 4-phosphate and NADPH. Our results showed that the expression of G6PD, tktA, PGLS, and PGD were up-regulated in shade-drying vs. fresh and smoke-drying vs. fresh; however, the expression of these proteins was down-regulated in shade-drying vs. smoke-drying. These results suggested that the drying process could significantly promote the PPP in rhubarb; however, the PPP was more active in smoke-drying rhubarb. It is well known that activated PPP synthesizes NADPH, helping maintain cell redox potential on the one hand. On the other hand, D-erythrose 4-phosphate synthesized by PPP acts as a substrate to synthesize active ingredients in rhubarb (Huang et al., 2016). Consistent with the present study, the PPP was found to be enhanced under salt (Huan et al., 2014), UV-B radiation (Zhao et al., 2015), drought (Landi et al., 2016), cold (Lin et al., 2013), and pathogen infection stress (Hu et al., 2019).

The glycolysis/gluconeogenesis pathway responds to biotic and abiotic stress, which affect ATP provision in plants (Zeng et al., 2019). In this study, the expression of phosphoglucose isomerase (GPI), fructose-bisphosphate aldolase (ALDO), glyceraldehyde-3 phosphate dehydrogenase (GAPDH), phosphoglycerate kinase (PGK), enolase (ENO), and pyruvate kinase (PK) were up-regulated in shade-drying vs. fresh and smoke-drying vs. fresh. However, the expression of these proteins was down-regulated in shade-drying vs. smoke-drying. PK, which is known to be a major rate-limiting enzyme in glycolysis, could act as a catalyzer for the last step of glycolysis during the conversion of phosphoenolpyruvate and ADP into ATP and pyruvic acid. Pyruvic acid also contributes to the creation of ATP based on the TCA cycle (Zeng et al., 2019). Amid the backdrop of shade-drying vs. fresh and smoke-drying vs. fresh, this protein was up-regulated; it is suggested that the promotion of PK expression may have generated more energy through the glycolysis pathway in order to tolerate the drying process. The expression of PK was down-regulated in shade-drying vs. smoke-drying, suggesting that the glycolysis pathway in smoke-drying was more active than in the shade-drying process.

The TCA cycle is the critical metabolic pathway allowing organisms to carry out energy metabolism, including the metabolism of the three main nutrients: sugars, lipids, and amino acids. During this process, the TCA cycle consumes pyruvate, reduces NAD^+^ to NADH, generates usable chemical energy in the form of ATP, and releases carbon dioxide (Fernie et al., 2004). In the current study, the DAP involvement in the TCA cycle was elevated when rhubarb was placed under the shade-drying vs. fresh or smoke-drying vs. fresh [e.g., aconitate hydratase (ACO), citrate synthase (CS), isocitrate dehydrogenase (IDH1), 2-oxoglutarate dehydrogenase complex (1 DLST), succinyl-CoA ligase (LSC1), fumarate hydratase 1 (fumC), malate dehydrogenase (MDH2)]. These findings indicated that shade-drying or smoke-drying processes promoted the TCA cycle in rhubarb. Enhanced TCA cycle and glycolytic activity increase the energy supply and carbon skeletons for the biosynthesis of defense-related proteins in response to oxidation, heat/high temperature, water loss, and starvation (Wu et al., 2020). CS is a vital enzyme in the TCA cycle. It is known that the elevated expression of CS can significantly improve plant resistance (Wang Q. et al., 2012). ACO is reported to be very sensitive to ROS and critically regulates cellular ROS homeostasis (Navarre et al., 2000; Moeder et al., 2007). 2-Oxoglutarate dehydrogenase complex (1 DLST) is a vital enzyme regulating the TCA cycle and significantly controls the accumulation of a-ketoglutarate and NADPH (Condori-Apfata et al., 2019). In the present research, CS, ACO, and IDH1 were down-regulated in shade-drying vs. smoke-drying. These data suggest that the TCA cycle is more active during the smoke-drying process. This increased activity of the TCA cycle is likely to enhance the accumulation of a-ketoglutarate, which will be involved in the synthesis of anthraquinones as important substrates.

As demonstrated previously, the rhubarb-drying process promotes starch and sucrose metabolism, the pentose phosphate pathway, glycolysis/gluconeogenesis, and the TCA cycle. First, the activation of the mentioned pathways provides rhubarb cells with sufficient ATP and NADPH to cope with the abiotic stress encountered during the drying process. Second, the accumulated intermediate products (e.g., a-ketoglutarate and D-erythrose 4-phosphate) create the material basis for the synthesis of anthraquinone and phenolic acids in rhubarb. Third, smoke-drying has a more significant up-regulatory effect on these pathways than shade-drying. It is worth noting that the increased respiration of rhubarb during the drying process will consume O_2_ and produce CO_2_. However, the smoke-drying process also depletes O_2_ to produce CO_2_. Therefore, a low concentration of O_2_ and a high concentration of CO_2_ may be one of the main factors affecting the accumulation of medicinally effective components in rhubarb.

### Proteins Related to the Synthesis of Anthraquinone and Phenolic Acids

In rhubarb, anthraquinone and phenolic acids act as the main active ingredients (Agarwal et al., 2001; Krafczyk et al., 2008). As reported previously, the shikimate pathway and the polyketide pathway are the main biosynthetic pathways leading to the production of anthraquinones in higher plants (Han et al., 2001; Perassolo et al., 2011). There are two main ways for the accumulation of anthraquinone substances in plants. One is that chorismate from the shikimate pathway and ketoglutarate from the TCA pathway undergo a series of reactions to finally synthesize anthraquinones (shikimic acid/o-succinylbenzoic acid pathway). The other is that malonyl CoA and acetyl-coenzyme A act as substrates to synthesize anthraquinone substances as impacted by polyketolase (polyketone pathway). Moreover, flavonoid biosynthesis critically impacts the production of phenolic acids (Wang Y. S. et al., 2012). In the present research, a total of 22 differential proteins related to the synthesis of active ingredients were identified in rhubarb undergoing different drying methods. In the present study, 12 and 10 differential proteins concerning the shikimate pathway and flavonoid biosynthesis, respectively, were identified during the rhubarb drying (Supplementary Table 4 and Figure 9).

The expression of enzymes related to the synthesis of active ingredients in rhubarb increased during the drying process; this finding was consistent with the results of content determination in shade-drying or smoke-drying for 4 days (Figure 1).

In plants, the shikimate pathway responds to differential environmental stresses (e.g., wounding, herbivore and microbial attack, or nitrogen starvation) (Weaver and Herrmann, 1997). Salt stress is known to promote shikimate dehydrogenase activity in wheat (Zeeshan et al., 2020), whereas chorismate synthase critically impacts barley–powdery mildew interactions (Hu et al., 2009). In the current study, the expressions of shikimate dehydrogenase (aroDE), 3-phosphoshikimate 1-carboxyvinyltransferase (aroA), and chorismate synthase (aroC) were significantly up-regulated in shade-drying vs. fresh and smoke-drying vs. fresh. Accordingly, the abiotic stress experienced during the drying process of rhubarb promoted the shikimic acid pathway. Moreover, the mentioned enzymes were found to be down-regulated in shade-drying vs. smoke-drying, proving that smoke-drying had a stronger promoting effect on the shikimic acid pathway and was more conducive to the accumulation of isochorismate, thus promoting the synthesis of anthraquinones.

The polyketide pathway is important in the synthesis of plant phenolic acid and resistance to abiotic stress (Bringmann et al., 2006; Cohen and Townsend, 2018; Abe, 2020). It is known that the chalcone synthase (CHS) gene is regulated by environmental stress (osmotic stress, UV stress, and pathogen stress) (Chen et al., 2015; Rodriguez-Calzada et al., 2019). Type III polyketide synthase and chalcone synthase were significantly up-regulated in shade-drying vs. fresh and smoke-drying vs. fresh. Furthermore, the mentioned enzymes were suggested to be down-regulated in shade-drying vs. smoke-drying, indicating that smoke-drying also had a stronger promotional effect on the polyketide pathway. Both anthocyanidin synthase (ANS) and anthocyanidin reductase (ANR) are closely related to the synthesis of phenolic acids in plants (Xie et al., 2003; Wellmann et al., 2006). Furthermore, ANS and ANR were significantly down-regulated in shade-drying vs. smoke-drying, proving that the smoke-drying process was more conducive to the accumulation of phenolic acids.

These results indicate that proteins concerned with phenolic acids and anthraquinone synthesis were up-regulated during the drying processes and expedited the synthesis and accumulation of the medicinally effective components in rhubarb. Moreover, the expression of the aforementioned proteins in the process of smoke-drying rhubarb was significantly higher than for shade-drying, revealing that smoke-drying caused a more significant accumulation of effective ingredients in the postharvest processing of rhubarb. This may be because smoke-drying rhubarb was subjected to heat/high temperature, hypoxia, and high concentrations of CO_2_.

## Conclusion

We set out to examine the molecular mechanisms involved in the accumulation of medicinally active ingredients in rhubarb during postharvest processing. Using proteomics, 1,927 DAPs were identified under different drying methods. Functional annotation demonstrated that many DAPs associated with the stress response and defense and stress signal transduction were up-regulated during the drying process of rhubarb; this indicated that rhubarb suffered severe stress during the drying process. We also found that the proteins related to the synthesis of active ingredients were significantly up-regulated during the drying process, and the expression of these proteins in smoke-drying rhubarb was higher than for shade-drying. The up-regulation of protein expression related to substance metabolism may be the main reason for the smoke-drying causing an improvement in the quality of rhubarb. These results indicate that the smoke-drying placed the harvested rhubarb under a strong abiotic stress environment (i.e., heat/high temperature, hypoxia, and high concentration of CO_2_), and this was more conducive to the accumulation of medicinally active ingredients. However, smoke-drying of rhubarb is a very complex process, and the main stress factors require greater examination in future research. In this article, the drying process of rhubarb was studied using proteomics for the first time; it provided a novel insight into the biosynthesis of active ingredients that occur during the rhubarb-drying process.

## Data Availability Statement

The datasets presented in this study can be found in online repositories. The names of the repository/repositories and accession number(s) can be found below: ProteomeXchange Consortium (http://proteomecentral.proteomexchange.org) via the iProX partner repository with the dataset identifier PXD024616.

## Author Contributions

YC, XL, WG, and FG designed the experiments. WL and JS analyzed the data and wrote the manuscript. XZ and BX commented on the manuscript. All authors read and approved the manuscript.

## Conflict of Interest

The authors declare that the research was conducted in the absence of any commercial or financial relationships that could be construed as a potential conflict of interest.

## Footnotes

1 http://www.geneontology.org

2 http://www.ncbi.nlm.nih.gov/COG

3 http://www.genome.jp/kegg
