# Supplementary material for: Label-Free Proteomic Analysis of Smoke-Drying and Shade-Drying Processes of Postharvest Rhubarb: A Comparative Study
Source: Front Plant Sci. 2021 May 26;12:663180. doi: 10.3389/fpls.2021.663180 (PMC8205111; doi:10.3389/fpls.2021.663180)
Supplement: Supplementary file 1 [file Data_Sheet_1.docx]

- **Label-free Proteomic Analysis of Smoke-drying and Shade-drying Processes of Postharvest Rhubarb: A Comparative Study**

**Wei Liang ^1^, Yuan Chen ^1*^, Xia Li ^2*^, Fengxia Guo ^1^, Jiachen Sun ^3^, Xuemin Zhang ^4^, Bo Xu ^4^ and Wenyuan Gao ^2*^**

1 Gansu Provincial Key Lab of Arid Land Crop Science, College of Agronomy, College of Life Science and Technology, Gansu Agricultural University, Lanzhou, China,

2 School of Pharmaceutical Science and Technology, Tianjin University, Tianjin, China,

3 School of Biotechnology and Food Science, Tianjin University of Commerce, Tianjin, China,

4 Key Laboratory of Modern Chinese Medicine Resources Research Enterprises, Tianjin, China

*** Correspondence:**Corresponding Author Yuan Chen, Xia Li, Wenyuan Gao
email@uni.edu *cygcx1963@163.com (Yuan Chen), lixia2008@tju.edu.cn (Xia Li), pharmgao@tju.edu.cn (Wenyuan Gao).*

Supplementary Material

**TableS1** Differentially abundant proteins related to signal transduction were partially selected in shade or smoke-drying rhubarb.

| Protein No. | Accession | Description | KO id | KO name | Log _2_ (Fold Change) | | | P_value | | | F_value | | |
| --- | --- | --- | --- | --- | --- | --- | --- | --- | --- | --- | --- | --- | --- |
|  |  |  |  |  | K-vs-X | Y-vs-X | K-vs-Y | K-vs-X | Y-vs-X | K-vs-Y | K-vs-X | Y-vs-X | K-vs-Y |
|  | MAPK signaling pathway - plant |  |  |  |  |  |  |  |  |  |  |  |  |
| 1 | TRINITY_DN10902_c0_g1_i3_orf1 | pathogenesis-related protein 1 | K13449 | PR1 | 1.2640 | 2.9130 | -1.6500 | 0.0214 | 0.0001 | 0.0002 | 6.9530 | 0.0371 | 187.3230 |
| 2 | TRINITY_DN5538_c0_g1_i1_orf1 | catalase | K03781 | CAT | 0.4457 | 2.1740 | -1.7280 | 0.0124 | 0.0003 | 0.0004 | 0.5497 | 1.0707 | 0.5134 |
| 3 | TRINITY_DN14743_c0_g1_i8_orf1 | PREDICTED: serine/threonine-protein kinase SRK2A | K14498 | SNRK2 | 7.4031 | 9.0709 | -0.1675 | 0.0000 | 0.0000 | 0.5585 | 0.0011 | 0.0009 | 1.2930 |
| 4 | TRINITY_DN971_c5_g1_i1_orf1 | PREDICTED: calmodulin | K02183 | CALM | -16.6100 | -16.6100 | 0.0000 | 0.0000 | 0.0000 | 1.0000 | 0.0000 | 1065.5089 | 0.0000 |
| 5 | TRINITY_DN40902_c0_g1_i1_orf1 | pathogenesis-related protein 1A-like | K13449 | PR1 | 1.4270 | 1.5230 | -0.0963 | 0.0002 | 0.0065 | 0.6448 | 0.4165 | 4.0564 | 0.1027 |
| 6 | TRINITY_DN10811_c0_g1_i1_orf1 | mitogen-activated protein kinase kinase | K20603 | MKK2 | 7.4031 | 9.0709 | -1.3440 | 0.0000 | 0.0000 | 0.0003 | inf | inf | 376.8135 |
| 7 | TRINITY_DN43280_c0_g1_i1_orf1 | unnamed protein product | K03781 | CAT | -16.6100 | -1.7520 | -16.6100 | 0.3546 | 0.0000 | 0.0000 | 1214.6810 | 1159.4108 | 1.0477 |
| 8 | TRINITY_DN971_c0_g2_i1_orf1 | calmodulin-7 isoform X3 | K02183 | CALM | -16.6100 | -1.7520 | -16.6100 | 0.0000 | 0.0453 | 0.0000 | 0.0000 | 1394.5580 | 0.0000 |
| 9 | TRINITY_DN1357_c0_g2_i1_orf1 | PREDICTED: nucleoside diphosphate kinase B | K00940 | NDK | 0.9789 | 1.9340 | -0.9548 | 0.0039 | 0.0001 | 0.0010 | 6.5261 | 1.3449 | 4.8524 |
| 10 | TRINITY_DN2275_c0_g1_i3_orf1 | nucleoside diphosphate kinase B | K00940 | NDK | 1.0660 | 1.5770 | -0.5119 | 0.0004 | 0.0001 | 0.0007 | 1.3095 | 0.0744 | 17.6083 |
| 11 | TRINITY_DN8006_c0_g2_i2_orf1 | nucleoside diphosphate kinase 2 | K00940 | NDK | 1.1060 | 1.5520 | -0.4456 | 0.0026 | 0.0004 | 0.0239 | 0.9812 | 0.4696 | 2.0896 |
| 12 | TRINITY_DN11583_c0_g2_i1_orf1 | Nucleoside diphosphate kinase | K00940 | NDK | 1.7130 | 2.0210 | -0.3079 | 0.0001 | 0.0022 | 0.1518 | 0.1107 | 1.3340 | 0.0830 |
| 13 | TRINITY_DN10902_c0_g1_i3_orf1 | pathogenesis-related protein 1 | K13449 | PR1 | 1.2640 | 2.9130 | -1.6500 | 0.0214 | 0.0001 | 0.0001 | 6.9530 | 0.0371 | 187.3230 |
| 14 | TRINITY_DN5538_c0_g1_i1_orf1 | catalase | K03781 | CAT | 0.4457 | 2.1740 | -1.7280 | 0.0124 | 0.0003 | 0.0004 | 0.5497 | 1.0707 | 0.5134 |
|  | Phosphatidylinositol signaling system |  |  |  |  |  |  |  |  |  |  |  |  |
| 15 | TRINITY_DN5098_c0_g1_i2_orf1 | inositol monophosphatase family protein | K15422 | SAL | 1.0360 | 1.7770 | -0.7418 | 0.0171 | 0.0001 | 0.0060 | 1.8115 | 0.0415 | 43.6318 |
| 16 | TRINITY_DN971_c5_g1_i1_orf1 | PREDICTED: calmodulin | K02183 | CALM | -16.6100 | -16.6100 | 0.0000 | 0.0000 | 0.0000 | 1.0000 | 0.0000 | 1065.5089 | 0.0000 |
| 17 | TRINITY_DN16794_c0_g1_i4_orf1 | inositol monophosphatase | K10047 | VTC4 | 0.7698 | 1.7970 | -1.0280 | 0.0279 | 0.0009 | 0.0055 | 1.5703 | 0.8633 | 1.8190 |
| 18 | TRINITY_DN971_c0_g2_i1_orf1 | calmodulin-7 isoform X3 | K02183 | CALM | -16.6100 | -1.7520 | -16.6100 | 0.0000 | 0.0453 | 0.0000 | 0.0000 | 1394.5580 | 0.0000 |
|  | Plant hormone signal transducti |  |  |  |  |  |  |  |  |  |  |  |  |
| 19 | TRINITY_DN10902_c0_g1_i3_orf1 | pathogenesis-related protein 1 | K13449 | PR1 | 1.0360 | 1.7770 | -0.7418 | 0.0214 | 0.0001 | 0.0002 | 6.9530 | 0.0371 | 187.3230 |
| 20 | TRINITY_DN14743_c0_g1_i8_orf1 | PREDICTED: serine/threonine-protein kinase SRK2A | K14498 | SNRK2 | 7.4031 | 9.0709 | -0.1675 | 0.0000 | 0.0000 | 0.5585 | 0.0011 | 0.0009 | 1.2930 |
| 21 | TRINITY_DN40902_c0_g1_i1_orf1 | pathogenesis-related protein 1A-like | K13449 | PR1 | 1.4270 | 1.5230 | -0.0963 | 0.0002 | 0.0065 | 0.6448 | 0.4165 | 4.0564 | 0.1027 |
|  | ABC transporters |  |  |  |  |  |  |  |  |  |  |  |  |
| 22 | TRINITY_DN11847_c0_g1_i1_orf1 | PREDICTED: ABC transporter C family member 14-like isoform X1 | K05666 | ABCC2 | 7.4031 | 0.0000 | 6.6493 | 0.0000 | 1.0000 | 0.0000 | 0.0045 | 0.0000 | inf |

**TableS2** Differentially abundant proteins related to stress response and defense were partially selected in shade or smoke-drying rhubarb.

| Protein No. | Accession | Description | KO id | KO name | Log _2_ (Fold Change) | | | P_value | | | F_value | | |
| --- | --- | --- | --- | --- | --- | --- | --- | --- | --- | --- | --- | --- | --- |
|  |  |  |  |  | K-vs-X | Y-vs-X | K-vs-Y | K-vs-X | Y-vs-X | K-vs-Y | K-vs-X | Y-vs-X | K-vs-Y |
|  | Peroxisome |  |  |  |  |  |  |  |  |  |  |  |  |
| 1 | TRINITY_DN6023_c0_g2_i1_orf1 | isocitrate dehydrogenase [NADP], chloroplastic/mitochondrial | K00031 | IDH1 | 7.4031 | 9.0709 | -1.9790 | 0.0000 | 0.0000 | 0.0062 | inf | inf | 3.2862 |
| 2 | TRINITY_DN2405_c1_g2_i2_orf1 | isocitrate dehydrogenase | K00031 | IDH1 | 0.4276 | 2.4450 | -2.0170 | 0.0263 | 0.0012 | 0.0017 | 0.2489 | 1.3400 | 0.1857 |
| 3 | TRINITY_DN256_c0_g1_i15_orf1 | 3-ketoacyl-CoA thiolase 2, peroxisomal [Citrus clementina] | K07513 | ACAA1 | 1.0870 | 2.1530 | -1.0670 | 0.0268 | 0.0002 | 0.0042 | 2.4230 | 0.2830 | 8.5625 |
| 4 | TRINITY_DN4477_c0_g1_i5_orf1 | PREDICTED: 2-hydroxyacyl-CoA lyase | K12261 | HACL1 | 1.1630 | 1.9940 | -0.8303 | 0.0019 | 0.0056 | 0.0387 | 4.7153 | 15.9094 | 0.2964 |
| 5 | TRINITY_DN4784_c0_g1_i2_orf1 | long chain acyl-CoA synthetase | K01897 | ACSL | 0.5489 | 2.8240 | -2.2760 | 0.3978 | 0.0017 | 0.0038 | 6.3042 | 0.8007 | 7.8731 |
| 6 | TRINITY_DN1089_c0_g1_i2_orf1 | peroxisomal coenzyme A diphosphatase NUDT7 | K17879 | NUDT7 | 1.3500 | 1.5540 | -0.2039 | 0.0091 | 0.0074 | 0.2821 | 1.1574 | 0.0003 | 3353.8665 |
| 7 | TRINITY_DN792_c1_g1_i1_orf1 | hydroxymethylglutaryl-CoA lyase, mitochondrial-like | K01640 | HMGCL | 1.4800 | 2.0420 | -0.5621 | 0.0027 | 0.0213 | 0.2012 | 0.4148 | 2.4760 | 0.1675 |
| 8 | TRINITY_DN6172_c0_g1_i2_orf1 | PREDICTED: superoxide dismutase [Cu-Zn], | K04565 | SOD1 | 0.7689 | 1.1330 | -0.3640 | 0.0016 | 0.0006 | 0.0223 | 0.7059 | 0.8454 | 0.8350 |
| 9 | TRINITY_DN373_c0_g1_i8_orf1 | PREDICTED: superoxide dismutase [Cu-Zn] 2 | K04565 | SOD1 | -1.2700 | -0.2881 | -0.9814 | 0.0034 | 0.1264 | 0.0006 | 0.6383 | 0.1448 | 4.4081 |
| 10 | TRINITY_DN43280_c0_g1_i1_orf1 | unnamed protein product | K03781 | CAT | 0.4605 | -16.6100 | 6.6493 | 0.3546 | 0.0000 | 0.0000 | 1214.6810 | 1159.4108 | 1.0477 |
| 11 | TRINITY_DN1346_c0_g2_i5_orf1 | PREDICTED: peroxisomal membrane protein PEX14 isoform X2 | K13343 | PEX14 | 0.8726 | 1.0940 | -0.2221 | 0.0158 | 0.0141 | 0.4150 | 5.3203 | 7.7229 | 0.6889 |
|  | Ascorbate and aldarate metaboli |  |  |  |  |  |  |  |  |  |  |  |  |
| 12 | TRINITY_DN5722_c0_g2_i2_orf1 | GDP-D-mannose 3', 5'-epimerase | K10046 | GME | 0.1699 | 1.5450 | -1.3750 | 0.4742 | 0.0024 | 0.0044 | 2.9996 | 1.6561 | 1.8113 |
| 13 | TRINITY_DN16794_c0_g1_i4_orf1 | inositol-phosphate phosphatase | K10047 | VTC4 | 0.7698 | 1.7970 | -1.0280 | 0.7698 | 1.7970 | -1.028 | 0.0279 | 0.0008 | 0.0055 |
| 14 | TRINITY_DN1460_c1_g2_i2_orf1 | PREDICTED: probable L-ascorbate peroxidase 6 | K00434 | APX | 0.9978 | 1.5000 | -0.5025 | 0.0005 | 0.0010 | 0.0207 | 0.8262 | 2.3688 | 0.3488 |
|  | Glutathione metabolism |  |  |  |  |  |  |  |  |  |  |  |  |
| 15 | TRINITY_DN6023_c0_g2_i1_orf1 | isocitrate dehydrogenase [NADP], chloroplastic/mitochondrial | K00031 | IDH1 | 7.4031 | 9.0709 | -1.9790 | 0.0000 | 0.0000 | 0.0062 | inf | inf | 3.2862 |
| 16 | TRINITY_DN2405_c1_g2_i2_orf1 | isocitrate dehydrogenase | K00031 | IDH1 | 0.4276 | 2.4450 | -2.0170 | 0.0263 | 0.0013 | 0.0017 | 0.2489 | 1.3400 | 0.1857 |
| 17 | TRINITY_DN1586_c0_g1_i7_orf1 | glutathione peroxidase | K00432 | gpx | 1.0070 | 1.4190 | -0.4120 | 0.0028 | 0.0001 | 0.0143 | 4.6052 | 0.3447 | 13.3613 |
| 18 | TRINITY_DN1184_c0_g1_i2_orf1 | glutamate--cysteine ligase, chloroplastic | K01919 | gshA | 7.4031 | 9.0709 | -1.0130 | 0.0000 | 0.0000 | 0.0092 | 0.0009 | 0.0003 | 2.7879 |
| 19 | TRINITY_DN796_c0_g1_i18_orf1 | hypothetical protein CCACVL1_06646 | K00383 | GSR | 7.4031 | 9.0709 | -1.5290 | 0.0000 | 0.0000 | 0.1494 | inf | inf | 461.4431 |
| 20 | TRINITY_DN7753_c0_g1_i6_orf1 | PREDICTED: protein HUA2-LIKE 2-like | K00432 | gpx | 1.5990 | 2.3890 | -0.7899 | 0.0637 | 0.0012 | 0.1183 | 1.8314 | 683.8087 | 0.0027 |
| 21 | TRINITY_DN934_c0_g1_i1_orf1 | glucose-6-phosphate 1-dehydrogenase | K00036 | G6PD | 0.6164 | 2.2870 | -1.6710 | 0.1052 | 0.0003 | 0.0012 | 9.8394 | 1.5693 | 6.2701 |
| 22 | TRINITY_DN1586_c0_g1_i5_orf1 | glutathione peroxidase | K00432 | gpx | 1.7570 | 2.6330 | -0.8762 | 0.0001 | 0.0001 | 0.0001 | 0.0356 | 0.0664 | 0.5359 |
| 23 | TRINITY_DN796_c0_g3_i2_orf1 | glutathione reductase | K00383 | GR | 0.8805 | 2.9010 | -2.0210 | 0.0373 | 0.0033 | 0.0008 | 0.0004 | 0.0002 | 1.7016 |
| 24 | TRINITY_DN8634_c0_g1_i7_orf1 | PREDICTED: putative glutathione peroxidase 7, chloroplastic | K00432 | gpx | 0.9716 | 1.1610 | -0.1894 | 0.0028 | 0.0014 | 0.1806 | 0.4266 | 0.4109 | 1.0381 |
| 25 | TRINITY_DN2999_c0_g1_i3_orf1 | 6-phosphogluconate dehydrogenase, putative | K00033 | PGD | 0.0000 | 9.0709 | -16.6100 | 1.0000 | 0.0000 | 0.0000 | 0.0000 | inf | 0.0000 |

**TableS3** Differentially abundant proteins involved in starch, carbohydrate and energy metabolism were partially selected in shade or smoke-drying rhubarb.

| Protein No. | Accession | Description | KO id | KO name | Log _2_ (Fold Change) | | |  | P_value |  |  | F_value |  |
| --- | --- | --- | --- | --- | --- | --- | --- | --- | --- | --- | --- | --- | --- |
|  |  |  |  |  | K-vs-X | Y-vs-X | K-vs-Y | K-vs-X | Y-vs-X | K-vs-Y | K-vs-X | Y-vs-X | K-vs-Y |
|  | Starch and sucrose metabolism |  |  |  |  |  |  |  |  |  |  |  |  |
| 1 | TRINITY_DN15_c0_g1_i11_orf1 | Glucose-1-phosphate adenylyltransferase | K00975 | glgC | 7.4031 | 9.0709 | 0.3496 | 0.0000 | 0.0000 | 0.3571 | inf | inf | 3.8479 |
| 2 | TRINITY_DN2276_c0_g1_i7_orf1 | UTP--glucose-1-phosphate uridylyltransferase | K00963 | UGP2 | 1.1360 | 1.8640 | -0.7288 | 0.0003 | 0.0001 | 0.0014 | 4.5457 | 3.5658 | 1.2748 |
| 3 | TRINITY_DN17781_c0_g1_i6_orf1 | nudix hydrolase 14, chloroplastic isoform X3 | K18447 | NUDX14 | -0.5920 | 0.8164 | -1.4090 | 0.0043 | 0.0160 | 0.0042 | 0.9361 | 4.3827 | 0.2136 |
| 4 | TRINITY_DN4864_c0_g3_i1_orf1 | NTP_transferase domain-containing protein | K00975 | glgC | 7.4031 | 9.0709 | -1.6620 | 0.0000 | 0.0000 | 0.0088 | inf | inf | 2607.0312 |
| 5 | TRINITY_DN8464_c0_g1_i1_orf1 | granule bound starch synthase I | K13679 | WAXY | 1.9270 | 2.4720 | -0.5446 | 0.0190 | 0.0023 | 0.0460 | 0.0514 | 0.0152 | 3.3894 |
| 6 | TRINITY_DN777_c0_g1_i8_orf1 | Phosphoglucomutase isoform 1 | K01835 | pgm | 3.4090 | 2.0550 | 1.3540 | 0.1949 | 0.0024 | 0.3558 | 20.5241 | 0.5382 | 38.1366 |
| 7 | TRINITY_DN770_c0_g1_i3_orf1 | glucose-1-phosphate adenylyltransferase large subunit 1-like | K00975 | glgC | 3.1550 | 4.8240 | -1.6690 | 0.0001 | 0.0003 | 0.0003 | 0.1097 | 0.2031 | 0.5399 |
| 8 | TRINITY_DN10571_c0_g1_i1_orf1 | venom phosphodiesterase 2 | K01513 | ENPP1_3 | 2.0930 | 4.0070 | -1.9140 | 0.0145 | 0.0013 | 0.0037 | 0.1526 | 0.0820 | 1.8611 |
| 9 | TRINITY_DN777_c0_g1_i37_orf1 | Alpha-D-phosphohexomutase superfamily | K01835 | pgm | 2.6940 | 3.3810 | 0.6878 | 0.0040 | 0.0001 | 0.0115 | 0.0504 | 0.0025 | 19.8629 |
| 10 | TRINITY_DN3083_c0_g1_i2_orf1 | phosphoglucomutase, cytoplasmic | K01835 | pgm | 0.8774 | 2.1100 | -1.2320 | 0.0014 | 0.0003 | 0.0010 | 2.0164 | 2.4213 | 0.8328 |
| 11 | TRINITY_DN1384_c0_g1_i6_orf1 | granule-bound starch synthase 2, chloroplastic/amyloplastic | K00703 | glgA | 1.3530 | 2.7180 | -1.3650 | 0.1651 | 0.0001 | 0.0144 | 7.1589 | 0.1293 | 55.3600 |
| 12 | TRINITY_DN4864_c0_g1_i5_orf1 | PREDICTED: glucose-1-phosphate adenylyltransferase small subunit 2, chloroplastic | K00975 | glgC | 1.1340 | 2.3070 | -1.1730 | 0.0002 | 0.0004 | 0.0017 | 0.4199 | 1.9652 | 0.2137 |
| 13 | TRINITY_DN1306_c0_g1_i6_orf1 | alpha-1,4 glucan phosphorylase L isozyme, chloroplastic/amyloplastic isoform X2 | K00688 | PYG | 1.1680 | 2.3460 | -1.1780 | 0.0006 | 0.0001 | 0.0005 | 1.0890 | 0.8990 | 1.2113 |
| 14 | TRINITY_DN1668_c0_g3_i1_orf1 | PREDICTED: starch synthase 1, chloroplastic/amyloplastic | K00703 | glgA | 1.3750 | 1.3570 | 0.0172 | 0.1564 | 0.0057 | 0.9656 | 0.0021 | 0.0002 | 13.2663 |
| 15 | TRINITY_DN3909_c0_g1_i4_orf1 | starch branching enzyme | K00700 | GBE1 | 0.6969 | 2.1710 | -1.4740 | 0.0009 | 0.0009 | 0.0019 | 0.4178 | 11.3187 | 0.0369 |
| 16 | TRINITY_DN777_c0_g1_i2_orf1 | phosphoglucomutase, chloroplastic-like | K01835 | pgm | 2.8510 | 3.6810 | -0.8308 | 0.0018 | 0.0001 | 0.0089 | 1.2763 | 0.4266 | 2.9920 |
| 17 | TRINITY_DN4644_c0_g1_i4_orf1 | Glycogen/starch/alpha-glucan phosphorylase | K00688 | PYG | 0.1712 | 2.1820 | -2.0110 | 0.3519 | 0.0004 | 0.0006 | 3.8725 | 2.2993 | 1.6842 |
| 18 | TRINITY_DN17511_c0_g2_i6_orf1 | alpha-glucosidase | K01187 | malZ | -1.5710 | -0.2268 | -1.3440 | 0.0001 | 0.0014 | 0.0001 | 43.1437 | 1.8663 | 23.1169 |
| 19 | TRINITY_DN17511_c0_g2_i2_orf1 | alpha-glucosidase | K01187 | malZ | -1.6880 | -1.0950 | -0.5938 | 0.0004 | 0.0004 | 0.0368 | 11.1678 | 1.5722 | 7.1035 |
| 20 | TRINITY_DN25836_c0_g1_i1_orf1 | hypothetical protein POPTR_016G077500 | K01193 | INV | 1.2920 | 3.2690 | -1.9770 | 0.2059 | 0.0407 | 0.0677 | 1.1286 | 0.0025 | 447.8497 |
| 21 | TRINITY_DN6265_c0_g2_i1_orf1 | vacuole invertase | K01193 | INV | -0.7490 | 0.3984 | -1.1480 | 0.0021 | 0.0614 | 0.0040 | 1.9616 | 4.0715 | 0.4818 |
| 22 | TRINITY_DN27010_c0_g1_i5_orf1 | hexokinase-1-like isoform X1 | K00844 | HK | 0.0000 | 9.0709 | -16.6100 | 1.0000 | 0.0000 | 0.0000 | inf | inf | 14414.8050 |
| 23 | TRINITY_DN2099_c0_g1_i3_orf1 | hexokinase-1 | K00844 | HK | 7.4031 | 9.0709 | -2.4270 | 0.0000 | 0.0000 | 0.0021 | 0.0015 | 0.0005 | 3.0542 |
| 24 | TRINITY_DN6444_c0_g1_i1_orf1 | Fructokinase-2 like | K00847 | scrK | 0.7303 | 1.1570 | -0.4266 | 0.0006 | 0.0014 | 0.0211 | 0.3170 | 1.8267 | 0.1736 |
| 25 | TRINITY_DN6848_c0_g1_i2_orf1 | probable fructokinase-4-like | K00847 | scrK | 1.8560 | 1.8910 | -0.0355 | 0.0004 | 0.0007 | 0.7765 | 0.0704 | 0.1317 | 0.5344 |
| 26 | TRINITY_DN4454_c0_g2_i1_orf1 | PREDICTED: probable fructokinase-7 | K00847 | scrK | 7.4031 | 9.0709 | -0.9590 | 0.0000 | 0.0000 | 0.0502 | inf | inf | 18.9524 |
| 27 | TRINITY_DN146_c0_g2_i3_orf1 | Phosphoglucose isomerase (PGI) | K01810 | GPI | 0.0690 | 4.1390 | -4.0690 | 0.9097 | 0.0001 | 0.0001 | 740.7238 | 5.3472 | 138.5268 |
| 28 | TRINITY_DN754_c0_g1_i4_orf1 | PREDICTED: glucose-6-phosphate isomerase 1, chloroplastic | K01810 | GPI | 1.5560 | 2.7260 | -1.1700 | 0.0189 | 0.0016 | 0.0143 | 2.4577 | 0.9849 | 2.4953 |
|  | Pentose phosphate pathway |  |  |  |  |  |  |  |  |  |  |  |  |
| 29 | TRINITY_DN7764_c0_g1_i2_orf1 | transketolase | K00615 | tktA | 1.2170 | 2.7910 | -1.5750 | 0.0191 | 0.0007 | 0.0029 | 10.0179 | 2.9640 | 3.3799 |
| 30 | TRINITY_DN6665_c0_g1_i1_orf1 | uncharacterized protein LOC114289396 | K00616 | talA | 7.4031 | 9.0709 | 0.9030 | 0.0000 | 0.0000 | 0.0128 | inf | inf | 0.3521 |
| 31 | TRINITY_DN5300_c0_g1_i1_orf1 | PREDICTED: 6-phosphogluconate dehydrogenase | K00033 | PGD | 1.7320 | 3.1800 | -1.4470 | 0.0018 | 0.0002 | 0.0009 | 0.4265 | 0.2393 | 1.7825 |
| 32 | TRINITY_DN146_c0_g2_i3_orf1 | Phosphoglucose isomerase (PGI) | K01810 | GPI | 0.0690 | 4.1390 | -4.0690 | 0.9097 | 0.0001 | 0.0000 | 740.7238 | 5.3472 | 138.5268 |
| 33 | TRINITY_DN934_c0_g1_i1_orf1 | glucose-6-phosphate 1-dehydrogenase | K00036 | G6PD | 0.6164 | 2.2870 | -1.6710 | 0.1052 | 0.0003 | 0.0012 | 9.8394 | 1.5693 | 6.2701 |
| 34 | TRINITY_DN9466_c0_g1_i7_orf1 | PREDICTED: ribulose-phosphate 3-epimerase, chloroplastic | K01783 | rpe | 7.4031 | 0.0000 | 6.6493 | 0.0000 | 1.0000 | 0.0000 | inf | 0.0000 | inf |
| 35 | TRINITY_DN754_c0_g1_i4_orf1 | PREDICTED: glucose-6-phosphate isomerase 1, chloroplastic [Nicotiana sylvestris] | K01810 | GPI | 1.5560 | 2.7260 | -1.1700 | 0.0189 | 0.0016 | 0.0142 | 2.4577 | 0.9849 | 2.4953 |
| 36 | TRINITY_DN7511_c0_g1_i1_orf1 | probable 6-phosphogluconolactonase | K01057 | PGLS | 0.6050 | 1.0360 | -0.4317 | 0.0033 | 0.0006 | 0.0107 | 0.7474 | 0.8976 | 0.8327 |
| 37 | TRINITY_DN8125_c0_g1_i1_orf1 | probable ribose-5-phosphate isomerase | K01807 | rpiA | -0.3908 | 0.9169 | -1.3080 | 0.1879 | 0.0006 | 0.0028 | 79.0395 | 3.9681 | 19.9190 |
| 38 | TRINITY_DN6665_c0_g2_i1_orf1 | Transaldolase | K00616 | talA, talB | 7.4031 | 0.0000 | 6.6493 | 0.0000 | 1.0000 | 0.0000 | inf | inf | 0.0020 |
| 39 | TRINITY_DN2999_c0_g1_i3_orf1 | 6-phosphogluconate dehydrogenase, putative | K00033 | PGD | 0.0000 | 9.0709 | -16.6100 | 1.0000 | 0.0000 | 0.0000 | 0.0000 | inf | 0.0000 |
| 40 | TRINITY_DN1327_c0_g2_i8_orf1 | hypothetical protein Ahy_A10g047817 isoform A | K01783 | rpe | 0.9568 | 1.9950 | -1.0380 | 0.0989 | 0.0001 | 0.0079 | 1.7682 | 0.0132 | 134.1614 |
|  | Glycolysis / Gluconeogenesis |  |  |  |  |  |  |  |  |  |  |  |  |
| 41 | TRINITY_DN14767_c0_g2_i1_orf1 | PREDICTED: pyruvate kinase 1, cytosolic-like | K00873 | PK | 1.2250 | 2.5220 | -1.2960 | 0.0485 | 0.0045 | 0.0168 | 0.1879 | 0.1515 | 1.2400 |
| 42 | TRINITY_DN27010_c0_g1_i5_orf1 | hexokinase-1-like isoform X1 | K00844 | HK | 0.0000 | 9.0709 | -16.6100 | 1.0000 | 0.0000 | 0.0000 | inf | inf | 14414.8050 |
| 43 | TRINITY_DN1046_c0_g4_i1_orf1 | fructose-bisphosphate aldolase 3, chloroplastic | K01623 | ALDO | 0.6462 | 1.0200 | -0.3741 | 0.0127 | 0.0029 | 0.0659 | 1.2007 | 1.0873 | 1.1043 |
| 44 | TRINITY_DN11928_c0_g1_i1_orf1 | Triosephosphate isomerase | K01803 | TPI | 1.0930 | 1.6150 | -0.5226 | 0.0001 | 0.0005 | 0.0110 | 0.9118 | 3.2433 | 0.2811 |
| 45 | TRINITY_DN237_c1_g3_i1_orf1 | Pyruvate kinase | K00873 | PK | 0.8961 | 2.1540 | -1.2580 | 0.0040 | 0.0001 | 0.0003 | 0.9127 | 0.3497 | 2.6100 |
| 46 | TRINITY_DN22159_c1_g1_i1_orf1 | glyceraldehyde-3-phosphate dehydrogenase 1, cytosolic | K00134 | GAPDH | -16.6100 | 1.0500 | -16.6100 | 0.0000 | 0.0603 | 0.0000 | 1.1248 | 1.0976 | 1.0248 |
| 47 | TRINITY_DN5728_c0_g1_i12_orf1 | hypothetical protein SOVF_007410 | K00927 | PGK | 1.5390 | 1.7080 | -0.1693 | 0.0288 | 0.0003 | 0.5613 | 1.7112 | 0.0943 | 18.1415 |
| 48 | TRINITY_DN185_c0_g1_i7_orf1 | glyceraldehyde-3 phosphate dehydrogenase | K00134 | GAPDH | 1.1240 | 2.1710 | -1.0480 | 0.0010 | 0.00008 | 0.0007 | 1.2944 | 0.8127 | 1.5926 |
| 49 | TRINITY_DN5901_c0_g1_i1_orf1 | PREDICTED: pyruvate kinase isozyme A, chloroplastic | K00873 | PK | 0.0000 | 9.0709 | -16.6100 | 1.0000 | 0.0000 | 0.0000 | 0.0000 | inf | 0.0000 |
| 50 | TRINITY_DN21685_c0_g2_i2_orf1 | PREDICTED: 2,3-bisphosphoglycerate-independent phosphoglycerate mutase 1 | K15633 | gpmI | -16.6100 | -16.6100 | 0.0000 | 0.0000 | 0.0000 | 1.0000 | 0.0000 | 1.0522 | 0.0000 |
| 51 | TRINITY_DN3691_c0_g1_i2_orf1 | Glyceraldehyde-3-phosphate dehydrogenase, type I | K00134 | GAPDH | 1.2180 | 2.1110 | -0.8927 | 0.0014 | 0.0079 | 0.0407 | 0.1059 | 1.1449 | 0.0925 |
| 52 | TRINITY_DN146_c0_g2_i3_orf1 | Phosphoglucose isomerase (PGI) | K01810 | GPI | 0.0690 | 4.1390 | -4.0690 | 0.9097 | 0.0001 | 0.0001 | 740.7238 | 5.3472 | 138.5268 |
| 53 | TRINITY_DN2099_c0_g1_i3_orf1 | hexokinase-1 | K00844 | HK | 7.4031 | 9.0709 | -2.4270 | 0.0000 | 0.0000 | 0.0021 | 0.0015 | 0.0005 | 3.0542 |
| 54 | TRINITY_DN3360_c0_g1_i3_orf1 | PREDICTED: pyruvate kinase isozyme G, chloroplastic | K00873 | PK | 7.4031 | 9.0709 | -1.2190 | 0.0000 | 0.0000 | 0.0008 | 0.0001 | 0.0002 | 0.4515 |
| 55 | TRINITY_DN4069_c0_g1_i9_orf1 | PREDICTED: phosphoglycerate kinase | K00927 | PGK | 0.9635 | 1.7810 | -0.8173 | 0.0001 | 0.0003 | 0.0023 | 1.5400 | 6.3992 | 0.2407 |
| 56 | TRINITY_DN3643_c0_g1_i8_orf1 | PREDICTED: triosephosphate isomerase, chloroplastic | K01803 | TPI | 0.9419 | 1.0810 | -0.1399 | 0.0013 | 0.0011 | 0.3163 | 3.8586 | 4.3038 | 0.8965 |
| 57 | TRINITY_DN22159_c1_g4_i1_orf1 | glyceraldehyde-3-phosphate dehydrogenase 2, cytosolic | K00134 | GAPDH | 7.4031 | 0.0000 | 6.6493 | 0.0000 | 1.0000 | 0.0000 | 1.0802 | 1.1763 | 0.9183 |
| 58 | TRINITY_DN754_c0_g1_i4_orf1 | PREDICTED: glucose-6-phosphate isomerase 1 | K01810 | GPI | 1.5560 | 2.7260 | -1.1700 | 0.0189 | 0.0017 | 0.0143 | 2.4577 | 0.9849 | 2.4953 |
| 59 | TRINITY_DN14065_c0_g3_i2_orf1 | fructose-bisphosphate aldolase | K01623 | ALDO | -16.6100 | -16.6100 | 0.0000 | 0.0000 | 0.0000 | 1.0000 | 0.0000 | 178.3068 | 0.0000 |
| 60 | TRINITY_DN252_c0_g1_i2_orf1 | 1 phosphoglyceromutase | K15633 | gpmI | 1.1420 | 2.0070 | -0.8651 | 0.0047 | 0.0009 | 0.0101 | 2.9423 | 2.1721 | 1.3546 |
| 61 | TRINITY_DN185_c0_g1_i3_orf1 | glyceraldehyde-3 phosphate dehydrogenase | K00134 | GAPDH | 0.7476 | 2.2270 | -1.4790 | 0.0192 | 0.0001 | 0.0004 | 1008.2954 | 164.2806 | 6.1376 |
| 62 | TRINITY_DN634_c0_g2_i2_orf1 | Pyruvate kinase, cytosolic isozyme | K00873 | PK | 1.0250 | 2.1120 | -1.0870 | 0.0001 | 0.0001 | 0.0008 | 0.1386 | 1.4213 | 0.0975 |
| 63 | TRINITY_DN1987_c0_g4_i1_orf1 | Phosphofructokinase domain | K00850 | PFK | 7.4031 | 9.0709 | -0.4434 | 0.0000 | 0.0000 | 0.0361 | 0.0000 | 1.1986 | 0.0000 |
| 64 | TRINITY_DN10733_c0_g1_i2_orf1 | enolase 1 | K01689 | ENO | 2.0360 | 3.1690 | -1.1330 | 0.0247 | 0.0053 | 0.0315 | 0.1112 | 0.0844 | 1.3185 |
| 65 | TRINITY_DN1575_c0_g1_i1_orf1 | PREDICTED: enolase | K01689 | ENO | 0.9986 | 1.9970 | -0.9980 | 0.0007 | 0.0004 | 0.0022 | 5.3991 | 8.3167 | 0.6492 |
| 66 | TRINITY_DN237_c1_g3_i2_orf1 | pyruvate kinase 1, cytosolic | K00873 | PK | 0.2975 | 2.8270 | -2.5290 | 0.5989 | 0.0046 | 0.0058 | 2.0877 | 0.3611 | 5.7809 |
| 67 | TRINITY_DN3691_c0_g1_i5_orf1 | glyceraldehyde-3-phosphate dehydrogenase GAPCP1, chloroplastic-like | K00134 | GAPDH | -16.6100 | 0.9613 | -16.6100 | 0.0000 | 0.0559 | 0.0000 | 0.0000 | 567.9036 | 0.0000 |
| 68 | TRINITY_DN14065_c0_g2_i1_orf1 | Fructose-bisphosphate aldolase | K01623 | ALDO | 0.7216 | -1.4250 | 2.1470 | 0.0739 | 0.0782 | 0.0001 | 0.0068 | 0.0177 | 0.3862 |
|  | TCA cycle |  |  |  |  |  |  |  |  |  |  |  |  |
| 69 | TRINITY_DN6023_c0_g2_i1_orf1 | isocitrate dehydrogenase [NADP], chloroplastic/mitochondrial | K00031 | IDH1 | 7.4031 | 9.0709 | -1.9790 | 0.0000 | 0.0000 | 0.0062 | inf | inf | 3.2862 |
| 70 | TRINITY_DN2405_c1_g2_i2_orf1 | isocitrate dehydrogenase | K00031 | IDH1 | 0.4276 | 2.4450 | -2.0170 | 0.0263 | 0.0013 | 0.0017 | 0.2489 | 1.3400 | 0.1857 |
| 71 | TRINITY_DN2877_c0_g1_i1_orf1 | 2-oxoglutarate dehydrogenase complex 1 | K00658 | DLST | 1.5250 | 2.2440 | -0.7193 | 0.0001 | 0.0001 | 0.0026 | 4.9486 | 18.2142 | 0.2717 |
| 72 | TRINITY_DN3972_c0_g1_i9_orf1 | Pyruvate dehydrogenase E1 component subunit beta-1 like | K00162 | PDHB | 1.2970 | 2.2320 | -0.9354 | 0.0001 | 0.0013 | 0.0090 | 7.8676 | 36.2290 | 0.2172 |
| 73 | TRINITY_DN753_c0_g2_i4_orf1 | malate dehydrogenase, chloroplastic | K00026 | MDH2 | 2.9150 | 2.3100 | 0.6050 | 0.0022 | 0.0997 | 0.1377 | 0.0002 | 0.0043 | 0.0470 |
| 74 | TRINITY_DN1604_c0_g1_i5_orf1 | hypothetical protein CISIN_1g001917mg | K01681 | ACO | 1.4760 | 3.4500 | -1.9750 | 0.0185 | 0.0077 | 0.0143 | 0.0323 | 0.1753 | 0.1844 |
| 75 | TRINITY_DN763_c0_g1_i3_orf1 | malate dehydrogenase, mitochondrial | K00026 | MDH2 | 0.7672 | 1.5800 | -0.8125 | 0.0014 | 0.0003 | 0.0020 | 1.0099 | 1.5090 | 0.6692 |
| 76 | TRINITY_DN925_c0_g1_i8_orf1 | PREDICTED: citrate synthase, glyoxysomal | K01647 | CS | 0.0014 | 2.2900 | -2.2910 | 0.9957 | 0.0001 | 0.0001 | 2.3092 | 0.0255 | 90.4453 |
| 77 | TRINITY_DN1557_c0_g1_i4_orf1 | hypothetical protein TanjilG_15952 | K01648 | ACLY | 1.4740 | 3.3580 | -1.8840 | 0.0136 | 0.0001 | 0.0003 | 0.3540 | 0.0499 | 7.0945 |
| 78 | TRINITY_DN18_c0_g1_i15_orf1 | ATP-citrate synthase | K01648 | ACLY | 0.8197 | 2.9280 | -2.1080 | 0.0085 | 0.0007 | 0.0012 | 3.0740 | 3.5582 | 0.8639 |
| 79 | TRINITY_DN5339_c0_g2_i10_orf1 | citrate synthase | K01647 | CS | 1.5700 | 2.5200 | -0.9498 | 0.0004 | 0.0008 | 0.0069 | 0.9619 | 2.2945 | 0.4192 |
| 80 | TRINITY_DN5862_c0_g1_i1_orf1 | hypothetical protein CCACVL1_26189 | K00234 | SDHA | 1.1690 | 2.9420 | -1.7730 | 0.0407 | 0.0010 | 0.0017 | 0.0329 | 0.0281 | 1.1700 |
| 81 | TRINITY_DN2476_c0_g2_i1_orf1 | dihydrolipoyllysine-residue acetyltransferase component 2 of pyruvate dehydrogenase complex, mitochondrial-like | K00627 | DLAT | 0.5539 | 1.4350 | -0.8812 | 0.0321 | 0.0011 | 0.0072 | 8.2705 | 5.2458 | 1.5766 |
| 82 | TRINITY_DN1559_c0_g2_i5_orf1 | PREDICTED: pyruvate dehydrogenase E1 component subunit alpha, mitochondrial | K00161 | PDHA | 1.3430 | 1.9410 | -0.5988 | 0.0003 | 0.0010 | 0.0203 | 2.5149 | 6.4596 | 0.3893 |
| 83 | TRINITY_DN14102_c0_g1_i1_orf1 | fumarate hydratase 1 | K01679 | fumC | 1.3710 | 2.2510 | -0.8791 | 0.0007 | 0.0008 | 0.0073 | 1.0243 | 1.9848 | 0.5161 |
| 84 | TRINITY_DN1039_c0_g2_i1_orf1 | isocitrate dehydrogenase [NAD] catalytic subunit 5, mitochondrial | K00030 | IDH3 | 1.1280 | 1.9150 | -0.7874 | 0.0239 | 0.0015 | 0.0266 | 1.6305 | 0.6567 | 2.4826 |
| 85 | TRINITY_DN1877_c0_g2_i1_orf1 | PREDICTED: isocitrate dehydrogenase [NAD] regulatory subunit 1, mitochondrial isoform X1 | K00030 | IDH3 | 1.7890 | 2.4970 | -0.7082 | 0.0001 | 0.0006 | 0.0097 | 0.0694 | 0.6134 | 0.1131 |
| 86 | TRINITY_DN5037_c0_g1_i1_orf1 | Succinyl-CoA ligase | K01899 | LSC1 | 1.3280 | 1.7980 | -0.4701 | 0.0054 | 0.0070 | 0.1586 | 66.5221 | 85.2291 | 0.7805 |
| 87 | TRINITY_DN4203_c0_g2_i1_orf1 | PREDICTED: dihydrolipoyllysine-residue acetyltransferase component 1 of pyruvate dehydrogenase complex, mitochondrial | K00627 | DLAT | 0.9449 | 2.1270 | -1.1820 | 0.0026 | 0.0008 | 0.0035 | 29.5314 | 43.3177 | 0.6817 |
| 88 | TRINITY_DN1877_c0_g1_i1_orf1 | PREDICTED: isocitrate dehydrogenase [NAD] regulatory subunit 1, mitochondrial | K00030 | IDH3 | 0.0000 | 9.0709 | -16.6100 | 1.0000 | 0.0000 | 0.0000 | 0.0000 | inf | 0.0000 |
| 89 | TRINITY_DN753_c0_g2_i2_orf1 | malate dehydrogenase | K00026 | MDH2 | 1.2670 | 1.8610 | -0.5944 | 0.0001 | 0.0001 | 0.0009 | 1.3427 | 0.7399 | 1.8146 |
| 90 | TRINITY_DN3972_c0_g1_i8_orf1 | hypothetical protein DM860_008887 | K00162 | PDHB | 1.6090 | 2.8160 | -1.2070 | 0.0009 | 0.0002 | 0.0014 | 0.3336 | 0.3533 | 0.9443 |
| 91 | TRINITY_DN2109_c0_g1_i1_orf1 | 2-oxoglutarate dehydrogenase complex 1 | K00658 | DLST | 0.3774 | 1.3970 | -1.0190 | 0.0056 | 0.0005 | 0.0010 | 0.3357 | 1.6351 | 0.2053 |
| 92 | TRINITY_DN4221_c0_g2_i1_orf1 | succinate--CoA ligase [ADP-forming] subunit beta, mitochondrial | K01900 | LSC2 | 1.0810 | 1.5010 | -0.4204 | 0.0001 | 0.000 | 0.0099 | 0.2841 | 0.8688 | 0.3270 |
| 93 | TRINITY_DN1498_c0_g1_i1_orf1 | PREDICTED: dihydrolipoyl dehydrogenase, mitochondrial | K00382 | DLD | 0.9123 | 2.3390 | -1.4270 | 0.0018 | 0.0007 | 0.0021 | 15.5201 | 28.8865 | 0.5373 |
| 94 | TRINITY_DN7320_c0_g3_i1_orf1 | putative aconitate hydratas | K01681 | ACO | 1.0080 | 2.9160 | -1.9080 | 0.0007 | 0.0016 | 0.0030 | 2.0329 | 10.8343 | 0.1876 |
| 95 | TRINITY_DN3972_c0_g1_i10_orf1 | Pyruvate dehydrogenase E1 component subunit beta-1 like | K00162 | PDHB | 0.6462 | 1.6180 | -0.9720 | 0.0061 | 0.0002 | 0.0013 | 8.1454 | 5.0825 | 1.6027 |
| 96 | TRINITY_DN1604_c0_g2_i1_orf1 | aconitate hydratase | K01681 | ACO | 1.1800 | 2.9050 | -1.7250 | 0.0015 | 0.00002 | 0.0001 | 0.3178 | 0.1316 | 2.4148 |
| 97 | TRINITY_DN2476_c0_g3_i1_orf1 | PREDICTED: dihydrolipoyllysine-residue acetyltransferase component 2 of pyruvate dehydrogenase complex, mitochondrial-like | K00627 | DLAT | 1.0490 | 1.7430 | -0.6943 | 0.0028 | 0.00003 | 0.0015 | 0.7641 | 0.0906 | 8.4356 |
| 98 | TRINITY_DN5245_c1_g1_i4_orf1 | dihydrolipoyllysine-residue acetyltransferase component 5 of pyruvate dehydrogenase complex, chloroplastic-like | K00627 | DLAT | 1.2890 | 1.4940 | -0.2054 | 0.0002 | 0.0001 | 0.0389 | 0.2522 | 0.1726 | 1.4608 |
| 99 | TRINITY_DN3522_c0_g1_i2_orf1 | hypothetical protein COLO4_15876 | K00025 | MDH1 | 1.0370 | 1.5900 | -0.5532 | 0.0051 | 0.0004 | 0.0220 | 11.1806 | 4.6555 | 2.4016 |
| 100 | TRINITY_DN763_c0_g1_i2_orf1 | hypothetical protein CDL15_Pgr007501 | K00026 | MDH2 | 0.9709 | 1.8760 | -0.9045 | 0.0004 | 0.0002 | 0.0010 | 0.7500 | 1.3002 | 0.5768 |

**TableS4** Differentially abundant proteins related to the synthesis of anthraquinone and phenolic acids were partially selected in shade or smoke-drying rhubarb.

| Protein No. | Accession | Description | KO id | KO name | Log _2_ (Fold Change) | | | P_value | | | F_value | | |
| --- | --- | --- | --- | --- | --- | --- | --- | --- | --- | --- | --- | --- | --- |
|  |  |  |  |  | K-vs-X | Y-vs-X | K-vs-Y | K-vs-X | Y-vs-X | K-vs-Y | K-vs-X | Y-vs-X | K-vs-Y |
|  | Shikimate pathway | Phenylalanine, tyrosine and tryptophan biosynthesis |  |  |  |  |  |  |  |  |  |  |  |
| 1 | TRINITY_DN32372_c0_g2_i2_orf1 | Chorismate synthase | K01736 | aroC | 2.9520 | 3.6110 | -0.6597 | 0.0001 | 0.0001 | 0.0025 | 0.0034 | 0.0257 | 0.1319 |
| 2 | TRINITY_DN6643_c0_g1_i2_orf1 | Aspartate/other aminotransferase | K14454 | GOT1 | 1.1970 | 2.4120 | -1.2150 | 0.0006 | 0.0001 | 0.0002 | 1.4394 | 0.6911 | 2.0829 |
| 3 | TRINITY_DN19673_c0_g1_i2_orf1 | indole-3-glycerol phosphate synthase, chloroplastic-like isoform X1 | K01609 | trpC | 1.0240 | 1.1830 | -0.1583 | 0.0024 | 0.0005 | 0.2656 | 2.1549 | 0.9596 | 2.2456 |
| 4 | TRINITY_DN300_c0_g2_i1_orf1 | DHquinase_I domain-containing protein/Shikimate_DH domain-containing protein/Shikimate_dh_N domain-containing protein | K13832 | aroDE | 0.0000 | 9.0709 | -16.6100 | 1.0000 | 0.0000 | 0.0000 | 0.0000 | inf | 0.0000 |
| 5 | TRINITY_DN4137_c0_g3_i1_orf1 | phospho-2-dehydro-3-deoxyheptonate aldolase 1  3-deoxy-7-phosphoheptulonate synthase | K01626 | aroF | 4.9030 | 6.2310 | -1.3280 | 0.0017 | 0.0050 | 0.0048 | 0.0003 | 0.0006 | 0.4226 |
| 6 | TRINITY_DN1393_c1_g2_i5_orf1 | 3-phosphoshikimate 1-carboxyvinyltransferase | K00800 | aroA | 1.6370 | 2.5550 | -0.9184 | 0.0243 | 0.0835 | 0.1418 | 0.0008 | 0.0049 | 0.1646 |
| 7 | TRINITY_DN19039_c0_g1_i3_orf1 | PREDICTED: shikimate dehydrogenase | K13832 | aroDE | 7.4031 | 9.0709 | -2.1020 | 0.0000 | 0.0000 | 0.2403 | inf | inf | 52.4176 |
|  | Flavonoid biosynthesis |  |  |  |  |  |  |  |  |  |  |  |  |
| 8 | TRINITY_DN8648_c0_g1_i2_orf1 | flavanone-3-hydroxylase | K00475 | E1.14.11.9 | 3.4500 | 5.1310 | -1.6810 | 0.0040 | 0.0024 | 0.0098 | 0.1297 | 0.1381 | 0.9388 |
| 9 | TRINITY_DN0_c0_g4_i2_orf1 | chalcone synthase 2 | K00660 | CHS | 7.4031 | 0.0000 | 6.6493 | 0.0040 | 1.0000 | 0.0000 | inf | inf | 0.9697 |
| 10 | TRINITY_DN12349_c1_g1_i5_orf1 | anthocyanidin reductase | K21102; K08695 | ANR | 0.8805 | 2.0920 | -1.2120 | 0.0047 | 0.0028 | 0.0093 | 1.9897 | 4.5117 | 0.4410 |
| 11 | TRINITY_DN8648_c0_g1_i3_orf1 | flavones 3-hydroxylase | K00475 | F3H | 1.2120 | 2.1700 | -0.9579 | 0.0016 | 0.0001 | 0.0011 | 0.9747 | 0.4235 | 2.3014 |
| 12 | TRINITY_DN4873_c0_g1_i2_orf1 | anthocyanin synthase | K05277 | ANS | 2.5800 | 3.3520 | -0.7725 | 0.0002 | 0.0001 | 0.0023 | 0.3327 | 0.2187 | 1.5217 |
| 13 | TRINITY_DN0_c0_g3_i2_orf1 | Type III polyketide synthase | K00660 | CHS | 7.4031 | 9.0709 | -1.2710 | 0.0000 | 0.0000 | 0.0064 | inf | inf | 0.7183 |
| 14 | TRINITY_DN566_c0_g1_i1_orf1 | cinnamate 4-hydroxylase | K00487 | CYP73A | 7.4031 | 9.0709 | -2.2000 | 0.0000 | 0.0000 | 0.0101 | 0.0159 | 0.0010 | 16.3773 |
| 15 | TRINITY_DN5604_c1_g1_i1_orf1 | dihydroflavonol-4-reductase | K13082 | DFR | 3.1400 | 3.4450 | -0.3047 | 0.0000 | 0.0001 | 0.0051 | 0.0058 | 0.0991 | 0.0582 |
| 16 | TRINITY_DN0_c0_g2_i2_orf1 | chalcone synthase 1 | K00660 | CHS | 2.4590 | 3.2280 | -0.7686 | 0.0001 | 0.0001 | 0.0011 | 0.0553 | 0.0255 | 2.1700 |
| 17 | TRINITY_DN2119_c0_g1_i5_orf1 | chalcone isomerase | K01859 | E5.5.1.6 | 0.7049 | 1.6630 | -0.9576 | 0.0013 | 0.0001 | 0.0001 | 0.4556 | 0.2771 | 1.6444 |
